# Developing comprehensive perinatal quality of care instruments in Mexico: An inclusive, multidisciplinary, and culturally sensitive approach

**DOI:** 10.1371/journal.pone.0352347

**Published:** 2026-07-16

**Authors:** Jimena Fritz, Pablo Montero-Zamora, Cara B. Safon, Pablo Martínez-Garrido, Matthias Sachse-Aguilera, Hernán García, Paola Sesia, Raffaela Schiavon, Héctor Lamadrid-Figueroa

**Affiliations:** 1 Department of Perinatal Health, National Institute of Public Health (INSP), Cuernavaca, Morelos, México; 2 Department of Kinesiology and Health Education, University of Texas at Austin, Austin, Texas, United States of America; 3 Department of Health Law, Policy, and Management, School of Public Health, Boston University, Boston, Massachusetts, United States of America; 4 UNICEF Mexico, Mexico City, Mexico; 5 Secretariat of Health, Mexico City, Mexico; 6 Center for Research and Higher Studies on Social Anthropology, Mexico City, Mexico; 7 Independent Consultant, Mexico City, Mexico; Federal University of Rio Grande do Norte: Universidade Federal do Rio Grande do Norte, BRAZIL

## Abstract

Ensuring high-quality care during pregnancy, childbirth, postpartum, and the newborn period is essential for reducing maternal and neonatal morbidity and mortality. In Mexico, a significant gap exists in comprehensive, culturally relevant instruments to assess quality of care (QoC) across these critical stages. This study aimed to develop robust, context-specific instruments to evaluate key dimensions of maternal and neonatal care, including patient experience, clinical processes, and hospital infrastructure. Using a modified Delphi method, we conducted two national workshops with 78 unique national experts, including academic researchers, healthcare decision-makers, and maternal health advocates. In Workshop 1, 45 experts identified 1,162 QoC indicators, which our research team subsequently reduced to 521 through a systematization process. Workshop 2 engaged 62 experts–approximately 20% of whom had also participated in Workshop 1–who discussed and rated the 521 indicators. Based on these expert ratings, our research team conducted a subsequent systematization, which further reduced the list to 489 QoC indicators. The final 489 indicators were categorized into five core QoC domains (i.e., *Woman, Care Processes, Health Personnel, Infrastructure, and Supplies and Medications*) and used to develop three instruments (1) a *Semi-Structured Interview Guide* to explore women’s care experiences; (2) a *Childbirth Observation Instrument* to assess QoC during labor and immediate postpartum care, including sociodemographic details, facility characteristics, clinical processes, and adherence to best practices, and (3) a *Hospital Information Instrument* to gather data on infrastructure, personnel, supplies, medications, and service utilization. These instruments offer a rigorous, context-specific framework for evaluating maternal and neonatal QoC in Mexico, helping identify gaps, guide improvements, and promote health equity. They address a critical need by providing tailored tools with strong potential to inform practice and improve outcomes.

## Introduction

Quality of Care (QoC) is recognized as both a human right and a reproductive right [1]. To uphold these rights globally, the World Health Organization (WHO) has called for strengthening health systems [2], and to prioritize QoC throughout the childbirth continuum as a significant area for research and intervention [3,4]. Appropriate care during pregnancy, birth, and postpartum is essential for reducing obstetric and neonatal complications, in turn decreasing maternal and perinatal morbidity and mortality [5,6].

Quality of maternal and neonatal healthcare is not just about service availability and accessibility; it also encompasses maternal satisfaction, which is influenced by factors such as facility conditions, human resource adequacy, and interpersonal behavior [7]. It has been proposed that maternal QoC should be evaluated from two perspectives (1) the quality of the provision of care within the institution, and (2) the QoC as experienced by the patients [8]. This framework acknowledges that service use and outcomes are influenced not only by the QoC provided but also by the women’s experiences of that care.

In Mexico, although the prevalence of skilled birth attendance is high, this indicator alone is not sufficient to ensure high-quality care [9]. While it measures the presence of trained personnel during birth, it fails to account for other critical dimensions of care characterized by indicators such as the appropriateness of interventions, adherence to evidence-based practices, and the overall experience of care from the woman’s perspective. Furthermore, evidence shows that harmful practices, such as unnecessary cesarean sections and routine episiotomies, persist despite the presence of skilled attendants, indicating a gap between technical competence and actual QoC provided [10–12].

Existing tools to measure QoC often disregard cultural sensitivity and regional socio-economic and healthcare aspects. They tend to focus on technical and clinical indicators without capturing the full scope of QoC, including the interpersonal aspects that significantly impact women’s experiences during childbirth [3,13–15]. This oversight is particularly problematic in a country where disparities in healthcare access and quality are pronounced, especially among socially vulnerable populations [16,17].

The persistence of high maternal mortality rates in Mexico further underscores these gaps [18]. These rates suggest that the current QoC measures do not adequately identify or address the underlying issues contributing to poor outcomes. Furthermore, the ongoing use of harmful practices in Mexico–such as the Kristeller manoeuvre and uterine sweeping–along with outright disrespect and obstetric violence, highlights the need for more comprehensive tools that can evaluate the full spectrum of QoC, from clinical practices to the dignity and respect afforded to women [12].

The partial nature of existing QoC measures demonstrates the need for new, comprehensive, and culturally sensitive instruments tailored to local or regional contexts. Such instruments must go beyond simple technical measures and include an evaluation of women’s experiences and the interpersonal dynamics of care. By incorporating diverse viewpoints from societal actors involved in pregnancy, birth, and neonatal care, our long-term goal is to create a more inclusive and representative process for measuring QoC. This approach ensures measurement captures both technical and clinical aspects of care, as well as essential elements like respect and dignity during childbirth. By addressing both technical quality and interpersonal aspects of care, QoC measurement can lead to more effective interventions and policies that promote respectful, dignified, and high-quality maternal and neonatal health outcomes in Mexico. Our study aimed to develop a set of comprehensive and culturally sensitive instruments to assess the QoC during prenatal, birth, postpartum, and newborn care in Mexico.

## Methods

We conducted two national workshops in Mexico City between November 5, 2014, and January 28, 2016, to develop a comprehensive approach for assessing quality of care (QoC) during pregnancy, childbirth, postpartum, and newborn care. Our research team engaged and collaborated with 78 national experts–including academics, policymakers, and activists specializing in maternal and perinatal health. Participants were selected through purposive sampling to ensure representation from key institutions involved in childbirth care in Mexico, as well as key stakeholders who, at the time, had contributed to the topic from academic, research, advocacy, and policy-making perspectives. They were not necessarily specialists in quality-of-care measurement; rather, they were selected for their expertise and/or stakeholder roles in maternal, perinatal, and newborn care in Mexico, and the workshops were structured to focus discussion on quality-of-care domains and indicators. The workshops were conducted using a structured, participatory format. Each workshop combined brief presentations with facilitated group discussions and collaborative activities designed to encourage dialogue and consensus-building. A professional facilitation firm was engaged to moderate discussions and synthesize the results. Outputs included documented discussion notes, thematic summaries, and jointly developed recommendations that informed subsequent analyses and deliverables. Participants were provided with background materials in advance, including relevant literature, data summaries, and guiding questions to support the workshop discussions and activities. Ethical approval for this study was obtained from the National Institute of Public Health in Mexico. All participants in Workshops 1 and 2 provided written informed consent prior to participation.

Using a modified Delphi method [19], the first workshop (i.e., Workshop 1) involved collaborative discussions and consensus-building to develop a comprehensive definition of QoC. Expert participants (*n* = 45) emphasized the importance of timely, evidence-based practices while integrating ethical principles and intercultural perspectives. Following the definition of QoC and informed by the IOM framework, participants identified core QoC domains that would encompass recommended indicators for care [20]. The IOM dimensions used were (1) *effectiveness*–providing services based on scientific knowledge to all who could benefit and refraining from providing services to those not likely to benefit, (2) *efficiency***–**avoiding waste, including waste of equipment, supplies, ideas, and energy, (3) *safety*–avoiding injuries to patients from the care that is intended to help them, (4) *patient-centeredness*–providing care that is respectful of and responsive to individual patient preferences, needs, and values, and ensuring that patient values guide all clinical decisions, (5) *equity*–providing care that does not vary in quality because of personal characteristics such as gender, ethnicity, geographic location, and socioeconomic status, and (6) *timeliness*–reducing waits and sometimes harmful delays for both those who receive and those who give care.

Further, experts collaboratively identified and face-validated specific indicators within each QoC domain. Following Workshop 1, the research team systematized and refined these indicators based on their alignment with the core QoC domains and relevant scientific evidence supporting recommended best practices. This process was conducted through facilitated group discussions, during which participants assessed the relevance, clarity, and completeness of the domains and indicators based on their expertise. Background materials and guiding questions were provided to support these activities, and consensus was reached collectively. These additional domains were identified and validated through the same facilitated group discussions and consensus-building process described above, with the aim of capturing context-specific aspects of maternal and neonatal care not fully addressed by existing frameworks. This initial systematization process (i.e., 1^st^ Systematization) was performed using MindManager version 15.1.173 software. By “systematization,” we refer to a structured and iterative process used to organize, harmonize, and refine the initial set of indicators. This process involved merging overlapping indicators, modifying definitions to improve clarity and relevance, retaining indicators that aligned with the study objectives, and excluding those deemed redundant, insufficiently supported by evidence, or not applicable to the Mexican context. These decisions were made collaboratively by the research team, informed by input from workshop participants, and guided by criteria including scientific validity, feasibility, policy relevance, and contextual applicability. The initial set of indicators was derived from a review of the scientific literature and informed by existing international frameworks and guidelines. These indicators were further refined and contextualized through expert input, with some additional indicators generated de novo to address gaps specific to the Mexican context.

Following Workshop 1 and the 1^st^ Systematization process, a second workshop (i.e., Workshop 2) was conducted with 62 participants, approximately 20% of whom had also attended Workshop 1. During Workshop 2, participants accessed an online survey hosted on REDCap and completed the questionnaire individually during the in-person workshop. To facilitate discussion, participants were organized into groups to review the indicators and exchange perspectives; however, each participant independently entered their own ratings for each indicator using a 5-point Likert scale (1 = *irrelevant*, 2 = *slightly relevant*, 3 = *moderately relevant*, 4 = *very relevant*, and 5 = *indispensable*). Consensus and retention decisions were not based solely on mean Likert scores. Quantitative ratings were used as an initial screening tool to identify indicators with broad support or divergence. These results were complemented by qualitative input from facilitated group discussions, where participants were able to contextualize their ratings, raise concerns, and suggest refinements. Decisions regarding indicator retention were reached by consensus throughout the workshops. Final determinations were made through deliberative review, taking into account the distribution of scores, qualitative feedback, alignment with study objectives, feasibility of measurement, and relevance to the Mexican context. This mixed-methods approach was chosen to ensure both methodological rigor and contextual validity.

After Workshop 2, our research team calculated mean scores for each indicator and ranked them based on experts’ ratings. A second systematization—conducted by our research team—then followed (i.e., 2nd Systematization), involving internal discussions to determine retention criteria. These criteria were defined as follows: indicators with mean scores between 4 and 5 (i.e., very relevant to indispensable) were considered to have strong expert support and were retained, whereas those scoring between 2.5 and 3.99 (i.e., slightly to moderately relevant) were interpreted as having more mixed support and therefore underwent a final review or third systematization (i.e., 3rd Systematization). Indicators scoring below 2.5 were considered to have low support and were not retained. During this final systematization, research team members independently evaluated each indicator with mean scores between 2.5 and 3.99 using a dichotomous scale (1 = *retain*, 0 = *exclude*). Indicators retained through this process were subsequently categorized according to the core QoC domains previously identified by experts during Workshop 1.

Informed by the finalized domains and indicators identified and face-validated by experts, we developed three comprehensive QoC instruments tailored for cultural relevance in the Mexican context. Once the final set of indicators had been categorized into the five core QoC domains, the research team used them to structure the content of the three instruments. Indicators under the Woman domain informed the semi-structured interview guide; indicators under the Care Processes domain informed the childbirth observation instrument; and indicators under the Infrastructure, Supplies and Medications, and Healthcare Personnel domains informed the hospital information instrument. Thus, the final systematized indicator set served as the substantive basis for the organization and content of the three data collection tools. To ensure alignment with local clinical practices, these instruments were cross-validated against Mexican clinical guidelines and norms [21,22].

## Results

### Defining QoC

During Workshop 1, 45 experts (66% female) convened to establish a shared understanding of the study objectives. Participants included 15 representatives from the public health system or related government branches, 19 academic researchers from diverse disciplines (e.g., public health, sociology, and obstetrics), 9 representatives of non-governmental organizations, and 2 representatives from international intergovernmental organizations. The workshop was facilitated by two professional moderators, one male and one female. The agreed upon definition of QoC stood as: “Quality of care during the preconception stage, pregnancy, childbirth, postnatal and newborn care is those timely actions (at technical and relational level) which are provided to women (and/or the newborn) by healthcare providers and health service managers with competencies based in evidence and according to applicable national and international normativity and have the required and sufficient infrastructure and supplies, following the dimensions applicable to individual- and collective-level of care. It is essential that system and providers offer care according to human rights, ethical principles, and considering the intercultural perspectives of their patients.”

### Identifying QoC Domains and Indicators

Informed by the IOM dimensions [20], participants identified, and face-validated, five core domains that–according to expert participants–captured maternal and neonatal QoC in the Mexican context. These domains include (1) *Woman* – which addresses the woman’s overall experience and satisfaction with care, covering her physical, emotional, and psychological needs throughout prenatal, birth, postpartum, and newborn stages, (2) *Care Processes* – which examines the quality and sequence of care provided from prenatal visits through delivery and postpartum follow-up, (3) *Health Personnel* – focusing on the qualifications, performance, and interpersonal skills of healthcare providers, and their adherence to professional standards and evidence-based practices,; (4) *Infrastructure* – evaluating the adequacy and safety of physical facilities, equipment, and the overall environment of the care setting; and (5) *Supplies and Medications* – assessing the availability, quality, and appropriate use of medical supplies and medications. Finally, participants worked together and identified 1,162 indicators, that from their perspective and experiences were crucial to measure QoC during pregnancy, delivery, postpartum, and newborn periods.

### Indicators’ Systematization and Experts’ Face Validation

Following Workshop 1 and subsequent systematization, the initial 1,162 indicators were reduced to 521 for rating in Workshop 2.

This initial pool reflected the breadth of expert perspectives and was subsequently refined through consensus. Notably, none of the indicators under *Supplies and Medications* and only one under *Health Personnel* (i.e., “Receives work incentives?”) fell below a mean score of 4, highlighting the strong consensus among experts on the importance of the vast majority of proposed indicators.

**Fig 1:**
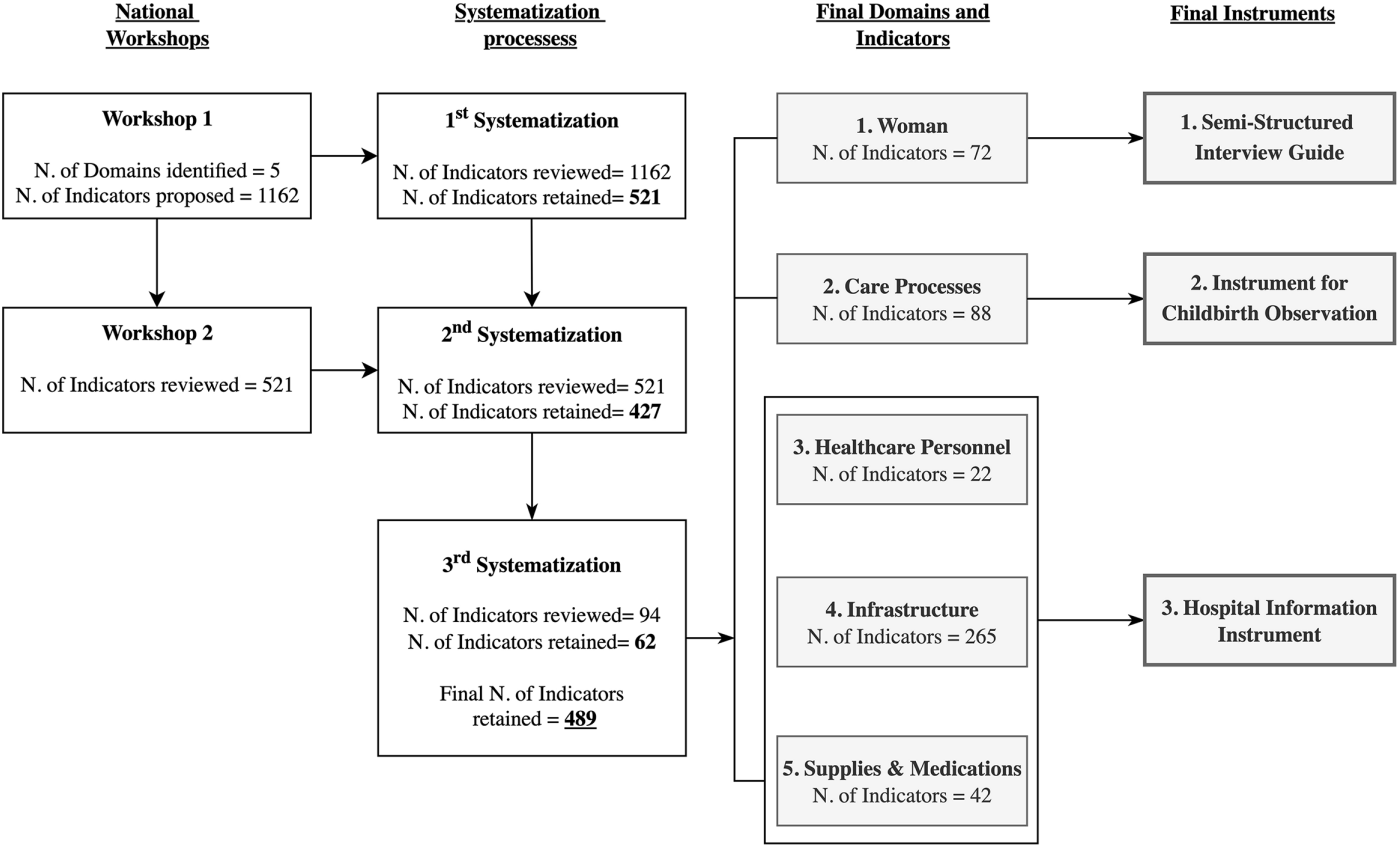
Identification and systematization of quality of care domains and indicators, and their categorization into final instruments.

Interestingly, some indicators scored exactly at the 2^nd^ Systematization’s inclusion threshold (mean = 4), indicating that indicators meeting the minimum consensus criteria were retained when they addressed critical gaps or covered important areas. Examples include satisfaction with family planning services, cleanliness of waiting room bathrooms, proper placement of evacuation routes and fire extinguishers, and the conditions of surgical or delivery rooms. Conversely, 94 (18.0%) indicators received mean scores between 2.5 and 3.99 (see Fig 1). These indicators were flagged for further discussion and subsequent review (i.e., 3^rd^ Systematization). Applying a more stringent inclusion criterion and following internal discussions within our research team, 62 of those 94 indicators were retained (~66%). These retained indicators received average scores of 0.5 or higher on a dichotomous scale (1 = *retain*, 0 = *exclude*). Many of them addressed issues such as emotional support during childbirth, postpartum hygiene counseling, and specific infrastructure or equipment considerations. Datasets containing the 521 reviewed indicators along with expert ratings are available for each of the five core QoC domains (S1–S5 Data).

The 3^rd^ Systematization process resulted in a final set of 489 QoC indicators. As shown in Fig 1, the final distribution included 72 under *Woman* (14.7%), 88 under *Care Processes* (18.0%), 22 under *Healthcare Personnel* (4.5%), 265 under *Infrastructure* (54.2%), and 42 under *Supplies and Medications* (8.6%). Throughout these multiple refinement steps, experts consistently prioritized *Infrastructure* and clinical *Care Processes*, reflecting the foundational role of facility readiness and labor management in quality maternal healthcare. The final distribution also shows a broader inclusion of woman-centered and supply-related indicators compared to earlier rounds, indicating a comprehensive approach balancing structural, procedural, and experiential dimensions. The rigorous revision of borderline indicators (i.e., mean scores < 4) highlights the expert panel’s commitment to including indicators that are not only relevant but also feasible and contextually appropriate for measuring QoC in Mexico. The final set of indicators used to construct the instruments is provided as Supporting Information (S6 Data).

### Top Consensus-Driven Indicators by QoC Domain

Experts identified key indicators for each core QoC domain. For the *Woman* perspective domain, the highest consensus was on the clarity of explanations regarding warning signs during prenatal consultations. Within the *Care Processes* domain, providing information to women about warning signs and symptoms was highly endorsed. In the *Healthcare Personnel* domain, experts emphasized the importance of measures for establishing and resolving complications at the hospital level. For the *Infrastructure* domain, the availability of secure ambulances for emergency transfers was prioritized. Lastly, the *Supplies and Medications* domain highlighted the necessity of having prenatal corticosteroids available in the hospital unit. The top three indicators for each QoC domain are presented in detail in Table 1.

**Table 1:** Top three consensus-driven indicators by quality of care domain.

Domain	Indicators*
**Woman**	Clarity of explanations regarding warning signs during pregnancy in prenatal consultations
	Conduct of vaginal examinations with appropriate explanation and dignity
	Clear communication of the benefits of breastfeeding and methods to facilitate it during postnatal consultations
**Care Processes**	Providing information to women about warning signs and symptoms
	Frequency of immediate breastfeeding after birth
	Identifying warning signs and symptoms in newborns during this period
**Healthcare Personnel**	Measures for the identification and management of complications at the hospital level (e.g., number of hysterectomies, obstetric haemorrhage, preeclampsia and eclampsia cases, and birth asphyxia).
	Attendance at vaginal deliveries
	Adherence to current clinical practice guidelines and pregnancy regulations
**Infrastructure**	Availability of secure ambulances or transportation for emergency transfers
	Telephone or radio equipment for communication with referral hospitals
	Presence and functionality of oxygen tanks in newborn care areas
**Supplies & Medications**	Presence of prenatal corticosteroids in the hospital unit
	Availability of uterotonics in the hospital unit
	Availability of anticonvulsant medications in the hospital unit

*Note: Indicators may be expressed as absolute counts or standardized as ratios (e.g., per number of deliveries), depending on data availability.

### Development of data collection instruments

As shown in Fig 1, the final set of indicators informed the development of three data collection instruments. The Semi-Structured Interview Guide explored women’s experiences during pregnancy, childbirth, postpartum, and postnatal care and was informed by the 72 indicators under the Woman domain (S1 Appendix; Spanish version in S4 Appendix). The Childbirth Observation Instrument documented and assessed QoC during childbirth, including hospital and sociodemographic information, details of the labor process, and adherence to best practices in childbirth and immediate postpartum care, and was informed by the 88 indicators under the Care Processes domain (S2 Appendix; Spanish version in S5 Appendix). The Hospital Information Instrument gathered data on hospital infrastructure, resources, health personnel, supplies, medications, and service utilization, and was informed by the 265 indicators under Infrastructure, 42 under Supplies and Medications, and 22 under Healthcare Personnel (S3 Appendix; Spanish version in S6 Appendix). The final version of the instruments was shared with participants and Mexican health authorities.

## Discussion

### Statement of principal findings

Our study identified five core domains and 489 indicators for measuring QoC in Mexico, resulting in the development of three comprehensive perinatal care quality instruments. These instruments provide a comprehensive approach which reflects the intention to capture multiple dimensions of quality of care, including clinical practices, health system capacity, and women’s experiences across the continuum of maternal and newborn care. This broad scope is also consistent with the clinical-indicator literature, which recognizes that quality may be assessed through indicators addressing structures, processes, and outcomes of care [23]. Their development represents an important step in operationalizing QoC as conceptualized by the expert group, both in defining QoC as established by the expert group and in developing comprehensive tools to effectively measure it within the Mexican healthcare system.

Findings reveal a strong consensus among experts on the critical elements of maternal and neonatal care, emphasizing the multiple dimensions of QoC. Key specific indicators identified include clear communication about warning signs, respectful women examination, managing hospital-level complications, guidelines adherence for care, and ensuring reliable emergency transportation. The need for essential medications like prenatal corticosteroids and uterotonics was also highlighted. The developed instruments are particularly significant because they are detailed and thorough, encompassing a wide range of maternal and child health-related domains and indicators. This comprehensiveness is crucial because, as Peter Drucker famously stated, “You can’t manage what you can’t measure” [24]. This level of detail is intended to support comprehensive assessment, recognizing that measurement is a prerequisite for informed quality improvement efforts, ultimately facilitating tailored and effective interventions for the Mexican context.

### Interpretation within the context of the wider literature

Measuring QoC across the continuum of pregnancy, childbirth, puerperium, and newborn care is a complex task, given the interaction of multiple factors, none of which is independent. We believe the Delphi method is the most appropriate for developing tools necessary for information gathering, integrating both qualitative and quantitative methodologies, and supporting multidisciplinary research. The Delphi method has been employed across various fields to determine process or outcome indicators [19,25,26]. Tripathi et al. used a modified Delphi method to develop and validate an index for assessing the quality of birth attendance [27]. By engaging global maternal and neonatal care experts, the researchers identified key dimensions and indicators of care quality. The final index consisted of 20 indicators and effectively distinguished between poorly and well-performed deliveries, later refined to 13 indicators for delivery-only assessment [28]. While these efforts focused on producing streamlined and validated tools suitable for rapid assessment, the objective of the present study was different and targeted improvements in facility-based deliveries, especially in resource-limited settings. However, while Tripathi’s objective was to create a concise 20-item index, our instruments aim to be much more comprehensive. The development of a reduced or composite index may represent a logical and valuable next step, following piloting and validation of the current instruments. This staged approach is also consistent with prior literature showing that, although numerous maternal and neonatal quality-of-care indicators have been proposed, relatively few have met all criteria for scientific soundness, underscoring the need for continued refinement, piloting, and validation of indicator-based measurement approaches [29].

The WHO defines QoC as the delivery of diagnostic and therapeutic services that are most appropriate for everyone to achieve optimal health outcomes, considering all factors and knowledge of the patient and medical service. This should result in the best possible health outcomes with minimal risk of iatrogenic effects and maximal patient satisfaction with the process [20,30]. Nevertheless, further empirical testing is required to determine how effectively these indicators capture QoC in practice, broken down into dimensions that are individually or collectively assessable, and considering women’s preferences and cultural perspectives.

It is important to recognize that patients play a fundamental role in identifying their own needs and preferences. Accordingly, one of the instruments developed in this study is a guide for semi-structured interviews with postpartum women. This is in line with the WHO’s emphasis on stakeholder involvement in health quality improvement [2,4]. Although the performance and acceptability of this instrument remain to be evaluated in real-world settings. The perspectives of women, families, and communities on the quality of maternity care services are crucial for informed decision-making, as noted by Kruk et al. [31] and are essential for increasing demand and access to quality services for women and newborns [32]. The instruments also incorporate a woman-centered and gender-responsive perspective by including indicators related to respectful and dignified care, effective communication, autonomy and informed decision-making, and women’s reported experiences of care. Together, these elements reflect a person-centered and rights-based approach to maternal and neonatal quality of care.

Furthermore, humanized childbirth and considering women at the center of pregnancy, childbirth, and postpartum care are important. The concept of “safe motherhood” often focuses narrowly on physical safety. However, the current instruments incorporate these dimensions conceptually, but their ability to measure such constructs reliably will require further validation, not only the technical practices and skills of health personnel but also their attitudes towards women and the way care is provided. This aligns with the WHO’s directive that health systems develop research on disrespect and assure high-quality, person-centered care [2,4].

Our approach resonates with recent WHO guidance on measuring and monitoring quality of care, which highlights the importance of selecting and analyzing fit-for-purpose QoC indicators to guide health system improvements and strengthen routine monitoring systems [33]. This alignment underscores the relevance of systematic indicator frameworks in both research and practice, as we did for the Mexican context.

### Implications for policy, practice, and research

The development of these instruments has significant implications for policy, practice, and future research. Policymakers can use these tools to develop more targeted interventions to improve maternal and neonatal health outcomes in Mexico (or any other country, after a proper validation). For healthcare providers, these instruments provide a structured approach to identifying specific areas for improvement, enhancing both patient care and overall service delivery. Future research should focus on piloting these instruments in diverse healthcare settings across Mexico to validate their effectiveness and refine their application. Additionally, exploring their applicability in other regions with similar healthcare challenges could enhance their utility on a broader scale. For this initiative, preliminary discussions with policymakers and public health stakeholders in Mexico have also been initiated to explore potential opportunities for piloting and implementing these instruments within the national health system.

### Strengths and limitations

A major strength of this study is its comprehensive approach for developing a unique QoC instruments. The modified Delphi method facilitated the integration of diverse expert insights, resulting in tools that are both culturally sensitive and relevant to the socio-economic and healthcare challenges in Mexico. These instruments, which are broad and detailed, encompassing a wide range of maternal and child health-related domains and indicators, were developed based on the health system context at the time of data collection and expert workshops. While core dimensions of quality of care are expected to remain relevant, health system organization, policies, and service delivery may evolve over time. Therefore, future piloting and validation efforts should consider recent and ongoing health system changes to ensure continued relevance and adaptability of the instruments.

A notable limitation of our study is that due to budgetary constraints, we were not able to properly pilot the developed instruments. We, however, decided to share these instruments with the scientific, and healthcare professional communities, with the hope that these will be tested in real-life settings, which could lead to further refinements. Second, Mexico’s vastness encompasses diverse cultural perspectives. Therefore, our instruments should be validated in different regions to ensure their representativeness and external validity, as well as potential further refinements following piloting. These instruments should be understood as a structured proposal for measurement rather than as validated tools ready for routine implementation. Finally, given the significant migration processes occurring in Latin America and through Mexico, future research should also assess the cultural relevance of these instruments among immigrants and migrants within this context.

Although the workshops informing the development of these instruments were conducted several years ago, the identified domains reflect core dimensions of maternal and newborn quality of care that remain relevant across health system contexts. Health system organization and service delivery conditions may evolve over time, but the principal contribution of this study lies in the identification and organization of foundational quality-of-care domains whose relevance extends beyond the specific context in which the workshops were conducted. Future piloting and application of these tools will allow their refinement and adaptation to evolving healthcare conditions, including recent health system changes and lessons from the COVID-19 pandemic.

## Conclusions

This study describes the development of QoC instruments for maternal and neonatal healthcare in Mexico through a collaborative, expert-driven process. The resulting tools are culturally and contextually tailored and provide a comprehensive framework for organizing and assessing key dimensions of QoC. These measures are intended to support future quality assessment efforts by offering a structured reference for evaluating patient-centeredness, safety, and equity across different care settings. However, they should be considered preliminary and require piloting and formal validation before informing implementation or policy decisions.

## Supporting information

S1 Appendix Semi-Structured Interview Guide for Women Users. (PDF)

S2 Appendix Childbirth Observation Instrument. (PDF)

S3 Appendix Hospital Information Instrument. (PDF)

S4 Appendix Semi-Structured Interview Guide for Women Users (Spanish Version). (PDF)

S5 Appendix Childbirth Observation Instrument (Spanish Version). (PDF)

S6 Appendix Hospital Information Instrument (Spanish Version). (PDF)

S1 Data Woman Quality of Care Domain: reviewed indicators and expert ratings. (ZIP)

S2 Data Care Processes Quality of Care Domain: reviewed indicators and expert ratings. (ZIP)

S3 Data Healthcare Personnel Quality of Care Domain: reviewed indicators and expert ratings. (ZIP)

S4 Data Infrastructure Quality of Care Domain: reviewed indicators and expert ratings. (ZIP)

S5 Data Supplies and Medications Quality of Care Domain: reviewed indicators and expert ratings. (ZIP)

S6 Data Final list of 489 quality-of-care indicators used to develop the instruments, with mean expert ratings. (ZIP)
