# Supplementary material for: Developing comprehensive perinatal quality of care instruments in Mexico: An inclusive, multidisciplinary, and culturally sensitive approach
Source: PLoS One. 2026 Jul 16;21(7):e0352347. doi: 10.1371/journal.pone.0352347 (PMC13374906; doi:10.1371/journal.pone.0352347)
Supplement: S1 Appendix — (PDF) [file pone.0352347.s001.pdf]

Project: "Quality of Care during Pregnancy, Childbirth, Postpartum, and for the Newborn (CAEPPyRN, acronym in Spanish) in Mexico"
ANNEX 1: Semi-structured Interview Guide for Users

Interviewer's Introduction

Thank you for your interest in participating in this interview. The objective of this interview is to gather the opinions of women who have recently given birth about the care they received during childbirth in the hospital.

This interview is part of a research study conducted by the National Institute of Public Health (INSP). It is important to understand that there are no right or wrong answers to the questions we will be asking. We are simply interested in hearing your opinions to help us make a proposal to improve the care provided at this hospital.

Before starting the interview, we will review a document that explains your rights as a participant in this study (informed consent). If you agree, we will ask you to sign the document. If you have any questions, please feel free to ask me at any time. [Obtain the participant's written consent and sign the copy of the form to record the signature and date.]

Participant Identification Number:

Date of the Interview:

Date of the Last Baby's Birth:

Interview Start Time:

1. GREETING AND GENERAL INFORMATION

Good morning or afternoon, how are you feeling today? To start, I would like to ask, how do you prefer to be addressed? Congratulations on your baby. Is it a boy or a girl? Does the baby have a name yet? What is the name

Let's start by talking about some general information:

1.1 Can you tell me how old you are? [Age:]

1.2 What is your level of education? (No education [1], primary school [2], secondary school [3], high school or technical school [4], university or higher education [5], No answer)

1.3 What is your marital status? (Married/Common-law [1], Single [2], Widowed/Divorced [3], Don't know/Not available [4])

1.4 What is your main occupation?

1.5 Who do you live with now?

1.6 How many pregnancies have you had? [Number:]

1.7 How many children do you have? [Number:]

1.8 Did any of your children were born by cesarean section? Yes [ ] No [ ]

1.9 Do you have any type of social security? Yes [ ] No [ ]
If so, which one? (IMSS, ISSSTE, Seguro Popular, Private Insurance)

1.10 Why did you choose to be treated at this hospital?

1.11 How long does it take to get to this hospital from your home?

1.12 Before becoming pregnant, did you visit a clinic to learn how to prepare for your pregnancy?
Yes [ ] No [ ]

2. EXPERIENCE DURING PREGNANCY

Let's talk a bit about your experience during pregnancy (for example, how it went for you and how you were treated).

2.1 Did anyone support you during your pregnancy? Yes [ ] No [ ] NR = No response [ ]
2.1.1Who?
2.1.2 How?

2.2 Did you attend prenatal check-ups? Yes [ ] No [ ] NR = No response [ ] If you did not attend, what was the reason? (Proceed to section 3)

2.3 If you did attend, could you tell me what your check-ups were like?
2.3.1 How many check-ups did you attend?
2.3.2 Where did you attend the check-ups?

2.3.3 Can you tell me who attended to you during your pregnancy? (General doctor, nurse, midwife, specialist, other)
2.3.4 On average, how long did you wait to enter your appointment(s)?
2.3.5 What do you think about the duration of the appointment(s)?
2.3.6 In general, during the appointments, were the explanations clear and understandable? Yes [ ] No [ ] NS = Don't know [ ] NR = No response [ ]

Do you remember what explanations were given to you? Can you give me some examples?

Did they ever listen to your:

Fears? Yes [ ] No [ ] NS = Don't know [ ] NR = No response [ ]

Expectations? Yes [ ] No [ ] NS = Don't know [ ] NR = No response [ ]

Opinions? Yes [ ] No [ ] NS = Don't know [ ] NR = No response [ ]

2.3.7 What do you think about the physical examination you received during the appointment(s)?
2.3.8 Were you prescribed any medications or vitamins during your pregnancy? Yes [ ] No [ ] NS = Don't know [ ] NR = No response [ ] (If no, proceed to 2.3.9)

2.3.8.1 If yes, where did you obtain them?

2.3.8.2 If yes, what medications or vitamins were prescribed to you?

2.3.9 Were the appointments free of charge? Yes [ ] No [ ] NR = No response [ ] If not, how much did you pay and where were they conducted?
2.3.10 Were you asked to undergo any laboratory tests? Yes [ ] No [ ] NR = No response [ ] (If no, proceed to 2.3.11)

2.3.10.1 If yes, where were the tests conducted?

2.3.10.2 If yes, what was the cost?

2.3.11 Did you ever feel discriminated against in the care you received during the appointments? Yes [ ] No [ ] NR = No response [ ] If yes, please explore the reasons why.
2.3.12 Were you informed about the warning signs during your pregnancy? (If no, proceed to 2.3.14) Yes [ ] No [ ] NR = No response [ ]
2.3.13 What do you think about the explanations and information provided by the healthcare staff regarding:

2.3.13.1 What’s was going to happen during childbirth?

2.3.13.2 The possible reasons for needing a cesarean section and what the consequences would be?

2.3.13.3 What would happen after your baby was born?

2.3.13.4 Contraceptives?

2.3.13.5 Personal care?

2.3.13.6 Breastfeeding?

2.3.13.7 Nutrition?

2.3.13.8 Vaccination? For your baby and for you

2.3.14 Did you and your doctor create a birth plan (or safety plan)? Yes [ ] No [ ] NR = No response [ ] (If no, proceed to section 3)

2.3.14.1 What did it consist of?

2.3.14.2 When you went to the hospital, was this plan followed? Yes [ ] No [ ] NR = No response [ ] If not, can you tell me why it could not be followed?

3. EXPERIENCE DURING CHILDBIRTH

Now let's talk about how your childbirth experience went.

3.1 Tell me about the transfer to get here.

3.2 How did you know it was time to go to the hospital? (Did you have contractions, mucus discharge, water breaking, etc.?)

3.3 How did you go to the hospital?

3.4 Who did you go with?

3.5 Were there any problems with the transfer? Yes [ ] No [ ] NR = No response [ ] What were they?

3.6 How much did you spend on the round-trip transfer?

3.7 Were you sent back from the hospital? Yes [ ] No [ ] NR = No response [ ] (If no, proceed to 3.8)

3.7.1 If yes, why?

3.7.2 What instructions were you given? (When to return, and in how much time)

3.8 How many times and who examined you before admitting you to the hospital?

3.9 How much dilation did you have upon admission?

3.10 Did you like how you were treated during admission? Yes [ ] No [ ] NS = Don't know [ ] NR = No response [ ]

Why?

4. EXPERIENCE OF CHILDBIRTH INSIDE THE HOSPITAL

Now let's talk about your labor experience inside the hospital.

4.1 Were you moved to the delivery room? Yes [ ] No [ ] NR = No response [ ] (If no, proceed to 4.3)

4.2 If yes, how many hours were you in the labor room with other women before being moved to the delivery room?

4.2.1 What do you think about that waiting time?

4.2.2 Where were you waiting to be attended to before delivery?

4.3 Do you think there were differences in the treatment of other women compared to yours? Yes [ ] No [ ] NR = No response [ ] Why do you think so?

4.4 Did someone accompany you most of the time during labor? Yes [ ] No [ ] NR = No response [ ] (If no, proceed to 4.5)

4.4.1 If yes, who were they?

4.4.2 If no, why not?

4.5 Were you asked to remove your clothing in front of other people? Yes [ ] No [ ] NR = No response [ ] If yes, how did you feel about it?

4.6 Were you clearly and understandably informed about what was happening and what was being done to you? Yes [ ] No [ ] NR = No response [ ]

4.7 Were you allowed to walk and move freely during labor? Yes [ ] No [ ] NR = No response [ ] Did you do so? Yes [ ] No [ ] NR = No response [ ]

4.8 Were you offered any liquids to drink? Yes [ ] No [ ] NR = No response [ ]

If yes, did you accept them? Yes [ ] No [ ] NR = No response [ ]

4.9 Were you shaved in the pubic area? Yes [ ] No [ ] NR = No response [ ]

4.10 Before your delivery, were you given an enema to clean your intestines? Yes [ ] No [ ] NR = No response [ ]

4.11 Were you given an intravenous drip (IV)? Yes [ ] No [ ] NS = Don't know [ ] NR = No response [ ]

4.12 Were you given medication to speed up the delivery of your baby? Yes [ ] No [ ] NR = No response [ ]

If yes, were you informed and explained why? Yes [ ] No [ ] NR = No response [ ]

4.13 Were you given an epidural anesthesia? Yes [ ] No [ ] NS = Don't know [ ] NR = No response [ ]

4.14 Were you asked what position you wanted to be in during contractions? Yes [ ] No [ ] NR = No response [ ] If yes, was it accommodated? Yes [ ] No [ ] NS = Don't know [ ] NR = No response [ ]

4.15 Were vaginal examinations performed on you? Yes [ ] No [ ] NR = No response [ ] (If no, proceed to 4.16)

4.15.1 If yes, how many times were they performed?

4.15.2 If yes, were you explained what it was about? Yes [ ] No [ ] NR = No response [ ]

4.16 Was your baby's heartbeat monitored with any device? Yes [ ] No [ ] NS = Don't know [ ] NR = No response [ ] (If no, proceed to 4.17)

If yes, were you explained why it was done? Yes [ ] No [ ] NR = No response [ ]

4.17 Were you offered any massage during labor?

Yes [ ] No [ ] NR = No response [ ] (If no, proceed to 4.18)

4.17.1 Was it done to you? Yes [ ] No [ ] NS = Don't know [ ] NR = No response [ ]

4.17.2 If yes, who performed it?

4.17.3 If yes, on which part of your body was it done?

4.17.4 How did you feel after the massage? Did it help?

4.18 Were you allowed to choose the position in which you felt most comfortable for your baby to be born? (If no, proceed to 4.19) Yes [ ] No [ ] NR = No response [ ]

4.18.1 In what position were you?

4.18.2 If no, had you requested it? Can you tell me more about why you were not allowed to?

4.19 Was your abdomen pressed to help the baby descend? Yes [ ] No [ ] NR = No response [ ]

4.20 Was an incision made to facilitate the baby's delivery (episiotomy)? Yes [ ] No [ ] NR = No response [ ]

Were you informed that this would be done? Yes [ ] No [ ] NS = Don't know [ ] NR = No response [ ]

4.21 After the placenta was delivered, was your entire hand inserted? (If no, proceed to 3.21) Yes [ ] No [ ] NR = No response [ ]

4.21.1 If yes, was anesthesia administered to your back? Yes [ ] No [ ] NR = No response [ ]

4.21.2 If yes, were you explained what this procedure was for? Yes [ ] No [ ] NR = No response [ ]

4.22 In general, throughout the entire care, do you feel you were treated with respect? Yes [ ] No [ ] NR = No response [ ]

4.23 Do you feel that you were given information during your pregnancy to make decisions during your delivery? Yes [ ] No [ ] NR = No response [ ]

Can you provide an example of this information? (If no, proceed to 4.25)

4.24 At the time of delivery, were you actually able to make any decisions? Yes [ ] No [ ] NR = No response [ ] (If no, proceed to 3.23.2)

Which ones?

4.25 In the final moments of delivery, when the baby was about to be born, did anyone support you? Yes [ ] No [ ] NR = No response [ ]

If yes, who was it and how did they assist?

4.26 Were you asked to sign any consent forms? Yes [ ] No [ ] NR = No response [ ] (If no, proceed to 4.27)

4.26.1 If yes, were you explained what it was for? Yes [ ] No [ ] NR = No response [ ]

4.26.2 If yes, did you agree? Yes [ ] No [ ] NR = No response [ ]

4.27 What did you like the most about your delivery? Why?

4.28 What did you like the least? Why?

4.29 Was there any problem during the delivery? Yes [ ] No [ ] NR = No response [ ]

What was it and how was it resolved?

4.30 Was the delivery room cold or warm? How did you feel about the temperature?

4.31 Will you or your family pay any amount for the delivery care? Yes [ ] No [ ] NS = Don't know [ ] NR = No response [ ]

If yes, how much will be paid?

4.32 Did you have to incur any expenses for medications, materials, or tests during delivery? Yes [ ] No [ ] NS = Don't know [ ] NR = No response [ ]

If yes, approximately how much did you spend?

5. EXPERIENCE IN THE IMMEDIATE POSTPARTUM

Now let's talk about your experience in the immediate postpartum period.

5.1 Was the baby taken away? Yes [ ] No [ ] NR = No response [ ] If yes, were you explained why and what they were going to do? Yes [ ] No [ ] NR = No response [ ]

5.2 Do you remember if the umbilical cord was cut immediately after birth or if they waited a bit?

5.3 Was the baby placed on your chest to breastfeed immediately after birth? Yes [ ] No [ ] NR = No response [ ]

5.4 Were you allowed to breastfeed the baby during the first hour? Yes [ ] No [ ] NR = No response [ ]

5.5 Did you experience any difficulty breastfeeding at that moment? Yes [ ] No [ ] NR = No response [ ] If yes, what was the difficulty and how did you resolve it?

5.6 Were you given any advice on how to breastfeed? Yes [ ] No [ ] NR = No response [ ] If yes, what was the advice and who gave it to you?

5.7 What do you think about the care your baby received?

5.8 Were you given information about contraceptive methods after delivery? Yes [ ] No [ ] NR = No response [ ]

5.9 Did you feel that any healthcare staff at the hospital pressured you to accept a contraceptive method? Yes [ ] No [ ] NR = No response [ ] If yes, what type of staff was it and what method were they trying to have you accept?

5.9.1 Did you choose any method? Yes [ ] No [ ] NR = No response [ ] If yes, which one?

5.9.2 Have you already received, had, or had it placed? Yes [ ] No [ ] NR = No response [ ]

5.10 ow long after the birth did they review you again? How was the review?

5.11 Did they give you any medication after the delivery? Yes [ ] No [ ] NR = No response [ ] If yes, what was it?

5.12 After your delivery, while you were recovering, did anyone support you? (If no, go to 5.13) Yes [ ] No [ ] NR = No response [ ]

5.12.1 Who?

5.12.2 How did they support you?

5.13 Were your family members given facilities to visit you during your hospitalization? [Yes [ ] No [ ] NS = Not sure [ ] NR = No response [ ]

5.14 After the delivery, how long were you told it would be until you were discharged?

5.15 Were your family members informed about your and your baby's health status after the delivery? Yes [ ] No [ ] NS= not sure [ ] NR= no response [ ]

5.16 Did they keep your baby with you the entire time after delivery? Yes [ ] No [ ] NR= no response [ ]

5.17 In general, did they consider any of your customs or advice from your family and/or acquaintances? (Drinking atole, walking, keeping the placenta...) Yes [ ] No [ ] NR= no response [ ] (If no, proceed to 5.18)

5.17.1 Which ones?

5.17.2 How?

5.17.3 If not, could you tell me more?

5.18 In total, from when you entered the hospital until now, how long have you been in the hospital?

6. EXPERIENCE IN POSTNATAL CONSULTATIONS (AFTER DISCHARGE)

Only in case the interview takes place during the postnatal consultation period.

6.1 On average, how long do you wait to be seen for your consultation(s)

6.1.1 How many consultations have you attended so far?

6.1.2 What do you think about the duration of the consultation(s)?

6.2 Were the benefits of breastfeeding explained to you clearly? Yes [ ] No [ ] NR=No response [ ]

6.3 Did anyone explain to you how to breastfeed your baby during a postnatal consultation? Yes [ ] No [ ] NR=No response [ ]"

6.2 Was a clear explanation given to you in any of the postnatal consultations about warning signs during the puerperium (42 days after delivery)? Yes [ ] No [ ] NR=No response [ ]"

6.3 Did the medical staff respect your decisions regarding contraception after delivery? Yes [ ] No [ ] NR=No response [ ]"

6.4 If you had an episiotomy during your delivery, have you experienced pain while urinating or during sexual intercourse as a result of this cut? Yes [ ] No [ ] NR=No response [ ]"

6.5 Were the prescriptions you received during the consultations complete? Yes [ ] No [ ] NR=No response [ ]"

6.6 Did you receive support during any of the postnatal visits?

Yes [ ] No [ ] NR=No response [ ] If yes, from whom and for what purpose."

6.7 Can you tell me how much time you spend on average for postnatal consultations, from the time you leave your house until you can return to your regular daily activities?"

6.8 What type of information and recommendations have you been given in the consultations after the birth of your baby to care for your health?

6.8.1 What have they been?

6.8.2 Who gave them to you and what have they been useful for? (e.g., breastfeeding, baby hygiene, warning signs for the baby's health, vaccination, screening, etc.)"

6.9 "If you had any complications after delivery, did the professional follow up on this during any postnatal consultation?

Yes [ ] No [ ] NR=No response [ ] (If no, skip to 6.12)

6.9.1 What was the complication?

6.9.2 How was it resolved?"

6.10 "Finally, during your pregnancy, delivery, and after delivery, have you experienced any form of mistreatment by any family member or health personnel?

Yes [ ] No [ ] NR=No response [ ] Can you explain in what way? (explore aspects of obstetric violence)"

CLOSING

Is there anything else you would like to add to this interview before we finish?

Thank you very much for your participation!

**References:**
1. Mexican Official Standard NOM-007-SSA2-2016, for the Care of Women During Pregnancy, Childbirth, and Postpartum, and of the Newborn.
2. Clinical Practice Guideline. Monitoring and Management of Labor in Low-Risk Pregnancy. Mexico: Ministry of Health; December 11, 2014.
3. Berdichevsky, K., Diaz-Olavarrieta, C., McCarthy, K., and Blanc, A. 2014. "Validating Indicators of the Quality of Maternal Health Care: Final Report, Mexico." Mexico City: Population Council.
4. National Institute of Public Health and Promoter Committee for Safe Maternity in Mexico. Results of the 1st Workshop: Quality of Care in Pregnancy, Childbirth, and Postpartum (CAEPP). November 5, 2014.
5. National Institute of Public Health. 2nd Workshop: Quality of Care in Pregnancy, Childbirth, Postpartum, and Newborn (CAEPPyRN). January 28, 2016.

6. Resource Model for Planning Health Units of the Ministry of Health (Humanized Birth Unit). General Directorate of Planning and Health Development (DGPLADES), Mexico 2016.

Collaborating Institutions and Participants in the CAEPPyRN Workshops

- San Miguel de Allende Adolescents Center, A.C. (CASA)
- Civic Collaboration Center (CCC)
- General Directorate of Planning and Health Development (DGPLADES)
- Institute of Security and Social Services for State Workers (ISSSTE)
- National Institute of Public Health (INSP)
- Health Secretariat of Morelos (SSM)
- IPAS, Mexico
- Promoter Committee for Safe Maternity in Mexico (CPMS)
- Mexican Social Security Institute (IMSS)
- Maternal Mortality Observatory (OMM)
- K'inal Antzetik, A.C.
- National Center for Gender Equity and Reproductive Health (CNEGySR)
- Quality Directorate, Health Services, Veracruz
- Center for Research and Higher Studies in Social Anthropology (CIESAS)
- National Institute of Perinatology (INPer)
- General Hospital of Tula, Health Services of Hidalgo (SSH)
- Empowered Maternity Collective (CME)
- Independent Consultant, Group for Information on Chosen Reproduction, A.C. (GIRE)
- Maternal-Infant Research Center of the Birth Studies Group (CIMIGEN)
- Research, Development, and Education Collective among Women, A.C. (CIDEM)
- FUNDAR, Center for Analysis and Research
- World Vision
- Save the Children
- Mexican Association of Midwifery (AMP)
- Luna Maya, Birth House
- Balance A.C.
- Health Secretariat of Durango (SSD)
- United Nations Children's Fund (UNICEF)
- Universidad de California, San Francisco (UCSF)
- Partners in Health
- MacArthur Foundation, México
