# Supplementary material for: Developing comprehensive perinatal quality of care instruments in Mexico: An inclusive, multidisciplinary, and culturally sensitive approach
Source: PLoS One. 2026 Jul 16;21(7):e0352347. doi: 10.1371/journal.pone.0352347 (PMC13374906; doi:10.1371/journal.pone.0352347)
Supplement: S2 Appendix — (PDF) [file pone.0352347.s002.pdf]

CAEPPyRN

Project: "Quality of Care in Pregnancy, Childbirth, Puerperium and the Newborn (CAEPPyRN) in Mexico".

ANNEX 2: Instrument for birth observation

Folio (number of delivery observed at this location): [ ]

Time of hospital admission: (hh:mm) |____|____| : |____|____|____|

Centimeters dilated at time of admission (check record):________________

Observation start time: (hh:mm) |____|____| : |____|____|____|

Dilation centimeters at observation start time (ask staff for information):___________________

Name of birth observer: ____

Date of observation ________/_________/________/ dd/mm/yyyy

**I. Hospital Information**
Name:
Clave Única de Establecimientos de Salud (CLUES):

**II. Women's Information**
**Instructions: The following information will be obtained by direct observation and from the pregnant woman's file. In those questions where a line appears write down what is being asked, in the other questions mark with an X in the Yes or No option.**

3. Date of birth: ____/____/____/ dd/ mm /yyyy	7. Previous vaginal deliveries: ________ (number)
4. Number of pregnancies:__________(numbers)	8. Previous cesarean sections: ________(number)
5. Gestational age:__________(weeks)	9. Previous abortions: ________ (number)

6. Is this a risky pregnancy? (see on file) YES: ___ No:___

6.1. What is the risk?____

IIa. Sociodemographic Information on Women.

Instructions: Record the appropriate number on the line.

10. Highest level of education completed by the woman: _____________________________

1. No schooling
2. Primary education
3. Secondary education
4. Higher secondary (Technical school and high school)
5. University or higher

88. Unknown/not available

11. Current marital status: ___________________________

1. Married / Common-law union
2. Single
3. Widowed / Divorced

88. No se sabe/no está disponible

12. Number of living daughters and sons currently: ___________________ (number)

13. Does the woman speak any indigenous language? (Observe or review her record) Yes: _______ No: _______

13a. What indigenous language does the woman speak? ____

14. If yes: Did any health staff address the woman in her language? (Observe if this occurs before, during, or after childbirth) Yes: _______ No: _______

III. Information about the person attending the delivery (who receives the baby).

Instructions: Review the record after the delivery to gather the necessary information and/or ask the healthcare provider. Note the letter or mark the corresponding option.

15. Ask for the age range; if not answered, note the apparent age: ( )

a) Under 20 years old   b) 20 to 24   c) 25 to 34   d) 35 to 44   e) 45 and more
16. Sex: Female: ( ) Male: ( )
17. Number of continuous hours worked before attending this delivery: ___________________________________
18. Profession: Write the letter of the corresponding profession in the parentheses. **( )**

a. Auxiliary nursing staff

b. General or technical nursing staff

c. Nursing staff with a bachelor's degree

d. Nursing and obstetrics staff (LEO)

e. Perinatal nursing specialist

f. Medical intern (undergraduate)

g. Medical intern in social service

h. General medical staff

i. Resident / specialist in gynecology

j. Resident / specialist in pediatrics

k. Medical staff in another specialty

l. Professional midwifery staff

(Nursing staff includes students and interns; verify the profession with the staff)

IV. Information about other personnel present during the delivery

Instructions: Mark with an X to indicate who was present during the delivery (expulsion period) and specify the number of men and the number of women for each profession.

Profession	**19. Present during the delivery (X = Yes)**	**19a Number of men**	**19b Number of women**
a)) General Nurse	( )	Number:______	Number:______
b) b) Bachelor of Nursing	( )	Number:______	Number:______
c) c) LEO (Bachelor of Nursing and Obstetrics)	( )	Number:______	Number:______
d) d) Perinatal Nurse	( )	Number:______	Number:______
e) e) Others (students, nursing interns)	( )	Number:______	Number:______
f) f) Medical Intern	( )	Number:______	Number:______
g) g) Medical Intern (social service)	( )	Number:______	Number:______
h) h) General Physician	( )	Number:______	Number:______
i) i) Resident or Specialist in Obstetrics and Gynecology	( )	Number:______	Number:______
j) j) Resident or Specialist in Pediatrics	( )	Number:______	Number:______
k) k) Physician with another specialty	( )	Number:______	Number:______
l) l) Others (students, medical interns)	( )	Number:______	Number:______
m) m) Professional Midwifery Staff	( )	Number:______	Number:______
n) n) Other (specify):________________________	( )	Number:______	Number:______

**Presence of a family member during the delivery**
o. Does a family member accompany the woman? **Yes: _______ No: _______**

p. If yes, specify which family member(s): ___________________________________

V. Dilatation period - First stage of labor

Instructions: Mark with an X to indicate if, no, or not applicable (NA)	YES	NO	NA
20. Was privacy provided to the woman (robe and/or curtains and/or doors)? At least one of the three elements must be present.			
21. Was the woman allowed to walk or sit (outside the bed) during labor?			
21a. Did the woman request this from the staff?			
22. Was the woman allowed to consume oral fluids during labor?			
22a. Did the woman request this from the staff?			
23. Is the woman catheterized?			
24. Was any medication administered to the woman?			
		go to p.26↓	go to p.26↓

24a. Mark with an X all the medications administered:

	( )
a. Antibiotic	( )
b. Analgesic	( )
c. Anticonvulsant	( )
d. Antihypertensive	( )
e. Magnesium Sulfate	( )
f. Corticosteroid for lung maturation	

Instructions: Mark with an X to indicate if, no, or not applicable (NA)	YES	NO	NA
25. Was epidural anesthesia administered during labor?			
26. Was non-pharmacological pain management promoted and/or facilitated during labor? (Such as walking, changing positions, massages, relaxation, breathing, etc.) Or a combination of any of these activities?			
		go to p.27↓	go to p.27↓
26a. If yes, which ones?			
27. Were warm compresses and/or perineal massage applied? (Care for perineal protection)			
28a. Specify the uterotonic and accumulated dose for the induction or conduction up to the time of observation (check the records): **Uterotonic:__________** **Dose:_________**		go to p.29↓	go to p.29↓
29. Was manual cervical dilation performed?			
30. Were manual perineal dilation maneuvers performed?			
31. Is a partogram used during labor?			
32. Was amniotomy performed?			

Instructions: Mark with an X to indicate if, no, or not applicable (NA)	YES	NO	NA

33. Did the healthcare staff mention the existence of abnormal labor progress (no cervical changes in 2 hours)?			
34. Did the healthcare staff mention any suspicion of fetal well-being issues or fetal distress?			
35. Was intermittent auscultation of fetal heart rate performed during labor (using a Pinard stethoscope or Doppler)?<br><br>If yes, note the time when each auscultation was performed:			
(hh:mm) |____|____| : |____|____| (hh:mm) |____|____| : |____|____| (hh:mm) |____|____| : |____|____| (hh:mm) |____|____| : |____|____| (hh:mm) |____|____| : |____|____| (hh:mm) |____|____| : |____|____|		**go to p.42↓**	**go to p.42↓**
42. Is continuous electronic monitoring performed to monitor fetal heart rate?			
43. Number of vaginal exams performed on the woman during labor:	Number:		
44. How many people?	Number:		
45. Did the staff ask the woman if she wanted to assume a special position to deliver her baby (sitting, standing, kneeling, semi-reclining, lateral position, or functional vertical birth)?			
		**go to p.47↓**	**go to p.47↓**

46. If yes, mark which ones:

a. Sitting	( )
b. Standing	( )
c. Kneeling	( )
d. Semi-reclining and/or lateral position	( )
e. Functional vertical birth	( )
f. Other. Which? ______________________________________	( )

VI. Expulsion Period - Second Stage of Labor

47. Time the observation of labor began : (hh:mm) |____|____| : |____|____|
(Time of admission to the delivery room)

Instructions: Mark with an X to indicate if, no, or not applicable (NA)	YES	NO	NA
48. Was the Kristeller maneuver (fundal pressure) performed?			
49. Was an episiotomy performed?			

VII. Placental Period - Third Stage of Labor

51. Time of birth of the baby : (hh:mm) |____|____| : |____|____|

52. Time of oxytocin administration : (hh:mm) |____|____| : |____|____|

53. Is oxytocin administered after the birth of the baby but before the delivery of the placenta?			
53b. What dose of oxytocin was administered? Dose:______________ (in units, IU)		go to p.54↓	go to p.54↓
54. Was the baby given to the mother immediately after birth?			
55. Was there immediate skin-to-skin contact between the mother and the newborn?			
55a. How long did they have skin-to-skin contact? Minutes:______________		go to p.56↓	go to p.56↓

56. Time of umbilical cord clamping: (hh:mm) |____|____| : |____|____|

57. At what time was the umbilical cord clamping performed ? ( )
(select only 1 option and indicate the letter in the parentheses)
a) Immediately after the baby's birth
b) Within the 1st minute after the baby's birth (between 11 seconds and 1 minute after birth)
c) After 1 minute of the baby's birth

Instructions: Mark with an X to indicate if, no, or not applicable (NA)	YES	NO	NA
58. ¿Se extrajo la placenta mediante tracción controlada o suave del cordón umbilical?			
59. ¿Se realizó contra-tracción del cordón umbilical?			

60. Hora del alumbramiento de la placenta: (hh:mm) |____|____| : |____|____|

61. ¿Realizaron masaje uterino (movimientos suaves de compresión en forma repetitiva con una mano en la parte inferior del abdomen de la mujer para estimular el útero)?			
		go to p.62↓	go to p.62↓

61a. At what time was the uterine massage performed? ( )
(select only 1 option and indicate the letter in the parentheses)
a) Before the expulsion of the placenta
b) After the expulsion of the placenta
c) Before and after the expulsion of the placenta

Instructions: Mark with an X to indicate if, no, or not applicable (NA)	YES	NO	NA
62. Were the placenta and its membranes examined after the birth of the baby?			
		go to p.64↓	go to p.64↓
63. Was the placenta and its membranes intact and normal?			
64. Is a manual examination of the uterine cavity performed?			
		go to p.66↓	go to p.66↓
65. Is an instrumental examination of the uterine cavity performed?			
Did they administer anesthesia/antibiotic/analgesia specifically for the examination of the uterine cavity?<br><br>What medication? _________________________ What dose? _______________<br>What medication? _________________________ What dose? _______________<br>What medication? _________________________ What dose? _______________		go to p.66↓	go to p.66↓
66. Did the delivery experience any complications?			
66a. What happened?		go to p.67↓	go to p.67↓
**VIII. After birth**			
67. Was the baby dried and stimulated within 30 seconds after birth?			
68. Were the baby's vital signs taken?			
68a. How many minutes after birth ?		go to p.69↓	go to p.69↓
69. Was Vitamin K given to the baby?			
70. Was ophthalmic antibiotic applied to the baby?			
71. Final time of birth observation : (hh:mm) |____|____| : |____|____| (time of leaving the expulsion room)			
72. Is breastfeeding observed within 60 minutes postpartum?			
73. Were the mother's vital signs taken?			
73a. How many minutes after birth?		go to p.74↓	go to p.74↓
**IX. General**			
74. Before performing any procedure during childbirth, was the woman explained clearly and understandably what was going to be done to her?			
75. Did the woman sign a consent letter for procedures in general?			
76. Final time of observation: (hh:mm) |____|____| : |____|____|			

Observations

**X. Write down any observations you deem appropriate regarding the observed delivery. If you left any question blank, or selected the option not applicable (NA), please write down the question number and explain why (for example: "the file was reviewed and the answer was not found or the action could not be observed because the woman arrived in her expulsion period").**
**References**

1. Mexican Official Standard NOM-007-SSA2-2016, For the care of women during pregnancy, childbirth and postpartum, and of the newborn.

2. Clinical Practice Guide. Surveillance and management of labor in low-risk pregnancy. Mexico: Ministry of Health; December 11, 2014.

3. Berdichevsky, K., Diaz-Olavarrieta, C., McCarthy, K., and Blanc, A. 2014. "Validating Indicators of the Quality of Maternal Health Care: Final Report, Mexico." Mexico City: Population Council.

4. National Institute of Public Health and the Committee for Safe Motherhood in Mexico. Results of the 1st Workshop: Quality of Care in Pregnancy, Childbirth and the Postpartum Period (CAEPP). November 5, 2014.

5. National Institute of Public Health. 2nd Workshop: Quality of Care in Pregnancy, Childbirth, Postpartum, and Newborns (CAEPPyRN). January 28, 2016.

6. Resource Model for the Planning of Medical Units of the Ministry of Health (Humanized Birth Unit). General Directorate of Planning and Development in Health (DGPLADES), Mexico 2016.

Collaborating institutions and participants in the CAEPPyRN Workshops:

- San Miguel de Allende Adolescents Center, A.C. (CASA)
- Civic Collaboration Center (CCC)
- General Directorate of Planning and Health Development (DGPLADES)
- Institute of Security and Social Services for State Workers (ISSSTE)
- National Institute of Public Health (INSP)
- Health Secretariat of Morelos (SSM)
- IPAS, Mexico
- Promoter Committee for Safe Maternity in Mexico (CPMS)
- Mexican Social Security Institute (IMSS)
- Maternal Mortality Observatory (OMM)
- K'inal Antzetik, A.C.

- National Center for Gender Equity and Reproductive Health (CNEGySR)
- Quality Directorate, Health Services, Veracruz
- Center for Research and Higher Studies in Social Anthropology (CIESAS)
- National Institute of Perinatology (INPer)
- General Hospital of Tula, Health Services of Hidalgo (SSH)
- Empowered Maternity Collective (CME)
- Independent Consultant, Group for Information on Chosen Reproduction, A.C. (GIRE)
- Maternal-Infant Research Center of the Birth Studies Group (CIMIGEN)
- Research, Development, and Education Collective among Women, A.C. (CIDEM)
- FUNDAR, Center for Analysis and Research
- World Vision
- Save the Children
- Mexican Association of Midwifery (AMP)
- Luna Maya, Birth House
- Balance A.C. - Balance A.C.
- Health Secretariat of Durango (SSD)
-United Nations Children's Fund (UNICEF)
-Universidad de California, San Francisco (UCSF)
-Partners in Health
-MacArthur Foundation, México
- San Miguel de Allende Adolescents Center, A.C. (CASA)
- Civic Collaboration Center (CCC)
- General Directorate of Planning and Health Development (DGPLADES)
