# Supplementary material for: Developing comprehensive perinatal quality of care instruments in Mexico: An inclusive, multidisciplinary, and culturally sensitive approach
Source: PLoS One. 2026 Jul 16;21(7):e0352347. doi: 10.1371/journal.pone.0352347 (PMC13374906; doi:10.1371/journal.pone.0352347)
Supplement: S3 Appendix — (PDF) [file pone.0352347.s003.pdf]

**Project: "Quality of Care in Pregnancy, Childbirth, Postpartum, and Newborn (CAEPPyRN) in Mexico"**

**ANNEX 3: Hospital Information Tool**

**Date of Application: _______/_______/_________ (dd/mm/yyyy)**
Day Month Year

**Name of the person completing the questionnaire:**_____________________________________________________
Name Surname

**Instrucciones: La información se obtendrá a través de observación en el sitio, revisión de libretas de labor, de sala de expulsión, de referencias y de entrevista con responsables de la unidad en el área médica (responsables de la dirección, subdirección y de las jefaturas de enfermería, enseñanza y epidemiología). Si existe información adicional relevante para el propósito de este trabajo, es posible realizar notas en los espacios en blanco de cada pregunta.**

| **1. GENERAL INFORMATION** | |
|---|---|
| 1.1 | Name of the institution: |
| 1.2 | CLUES (Unique Key for Health Facilities): |
| | d) Delegation/Municipality e) State f) Zip Code |

| a. Name and position of the person(s) providing the information: | | | |
|---|---|---|---|
| a1 | | | |
| | Name | Position | Surname |
| a2 | | | |
| | Name | Position | Surname |
| a3 | | | |
| | Name | Position | Surname |
| a4 | | | |
| | Name | Position | Surname |
| a5 | | | |
| | Name | Position | Surname |

| b. Specify the notebook(s) or document(s) from which the information was obtained: | |
|---|---|
| b1 | |
| b2 | |
| b3 | |
| b4 | |
| b5 | |

| Instructions: Review the following information in obstetrics or statistics notebooks. | | |
|---|---|---|
| 1.3 | Name of the main referral hospital: | |
| 1.4 | Does it have the name and phone number of the person responsible for managing transfers? | Yes………………. ( ) |
| | | No………………. ( ) ↓ **1.6** |
| 1.4a | **If yes, please note the details:** | |
| | **Name:** | |
| 1.4b | **Phone numer:** | |
| 1.5 | Time taken to reach the referral hospital by vehicle: | **Minutes:** |
| 1.6 | How many functional ambulances with fuel are available 24 hours a day? | **Number:** |
| 1.7 | Is there at least 1 driver available for the ambulance 24 hours a day? | **Number:** |
| 1.8 | In the case of a referral to another health facility, does the staff of this hospital provide or arrange transportation for the referral? | Yes………………. ( ) |
| | | No………………. ( )↓ ***Sec. 2*** |
| 1.9 | In the case of a referral, does the staff of this unit communicate with the hospital staff to inform them about the referral? | Yes………………. ( ) |
| | | No………………. ( ) |
| 1.10 | In the case of a referral, does the health staff accompany the | Yes………………. ( ) |

| Comments on Section 1 (Specify the question number and comment) |
|---|
| |
| |
| |
| |
| |
| |
| |
| |
| |

| | | |
|---|---|---|
| | woman? | No………………. ( ) |

| **2. INFRASTRUCTURE AND OTHER RESOURCES** | | | | | |
|---|---|---|---|---|---|
| **Instructions:** In the following questions, mark with an X "Yes" or "No" if the health facility currently has the specified equipment, supplies, or infrastructure. If the answer is "Yes," record the available number and the number of functioning units in the corresponding "Number:_______" boxes. If you have any observations for any of the questions, note them in the "Comments" section. | | | | | |
| **Hospital Area or Zone** | | **No** | **Yes** | **Available Units** | **Working Units** |
| **2.1 Laboratory Service** | | ( ) | ( ) | | |
| **2.2 Obstetric Imaging Service** | | ( ) | ( ) | | |
| **2.3 Obstetric Ultrasound Service** | | ( ) | ( ) | | |
| **2.4 Obstetric Admission Waiting Room** | | ( ) **↓2.5** | ( ) | | |
| 2.4.1 | Signage in the local language | ( ) | ( ) | | |
| 2.4.2 | Ramps for user Access | ( ) | ( ) | Number: | Number: |
| 2.4.3 | Appropriate and complete lighting | ( ) | ( ) | | |
| 2.4.4 | Evacuation routes and fire extinguishers placed in the appropriate locations (in accordance with NOM-002-STPS-2000) | ( ) | ( ) | | |
| 2.4.5 | Air conditioning | ( ) | ( ) | Number: | Number: |
| 2.4.6 | Area for Obstetric Triage | ( ) | ( ) | | |
| 2.4.7 | Are the spaces in good condition? (For example: walls without moisture, cracks, holes, water leaks, drips) | ( ) | ( ) | | |
| 2.4.8 | Men's restroom | ( ) **↓2.4.9** | ( ) | Number: | Number: |
| *2.4.8.1* | *Can the men's restrooms be used by patients and/or* | | | *Yes……………………. ( )* | |

| | | | | | |
|---|---|---|---|---|---|
| | *companions?* | | | *No......................... ( )* | |
| 2.4.8.2 | *Do the men's restrooms have physical spaces for access and use by people with disabilities?* | | | *Yes........................ ( )*<br>*No......................... ( )* | |
| 2.4.8.3 | *Do the men's restrooms have diaper changing stations?* | | | *Yes........................ ( )*<br>*No......................... ( )* | |
| 2.4.9 | Women's restroom | ( ) **↓2.5** | ( ) | Number: | Number: |
| 2.4.9.1 | *Can the women's restrooms be used by patients and/or companions?* | | | *Yes........................... ( )*<br>*No......................... ( )* | |
| 2.4.9.2 | *Do the women's restrooms have physical spaces for access and use by people with disabilities?* | | | *Yes........................ ( )*<br>*No......................... ( )* | |
| 2.4.9.3 | *Do the women's restrooms have diaper changing stations?* | | | *Yes........................... ( )*<br>*No......................... ( )* | |

| Hospital Area or Zone | | No | Yes | Available Units | Working Units |
|---|---|---|---|---|---|
| **2.5 MATER Module / Emergency Room** | | ( ) **↓2.6** | ( ) | | |
| 2.5.1 | Adequate lighting and ventilation | ( ) | ( ) | | |
| 2.5.2 | Space for handwashing (sink) | ( ) | ( ) | Number: | Number: |
| 2.5.3 | Chair for pregnant women | ( ) | ( ) | Number: | Number: |
| 2.5.4 | Chute lamp | ( ) | ( ) | Number: | Number: |
| 2.5.5 | Negatoscope | ( ) | ( ) | Number: | Number: |
| 2.5.6 | Examination table with legs | ( ) | ( ) | Number: | Number: |
| 2.5.7 | Screens or curtains to provide privacy to the user | ( ) | ( ) | Number: | Number: |
| 2.5.8 | Sphygmomanometer | ( ) | ( ) | Number: | Number: |
| 2.5.9 | Binaural stethoscope | ( ) | ( ) | Number: | Number: |
| 2.5.10 | Complete diagnostic kit | ( ) | ( ) | Number: | Number: |
| 2.5.11 | Scale | ( ) | ( ) | Number: | Number: |
| 2.5.12 | Stadiometer | ( ) | ( ) | Number: | Number: |
| 2.5.13 | Thermometer | ( ) | ( ) | Number: | Number: |
| 2.5.14 | Disposable vaginal mirror | ( ) | ( ) | Number: | Number: |
| 2.5.15 | Portable fetal heartbeat phonodetector (Doppler) | ( ) | ( ) | Number: | Number: |

| Hospital area or zone | | No | Yes | Available Units | Working Units |
|---|---|---|---|---|---|
| **2.6 Labor room** | | ( ) **↓2.7** | ( ) | Number: | |
| **The following questions apply to the total number of labor rooms in this hospital.** | | | | | |
| 2.6.1 | Clean (no moisture, cracks, holes in ceilings and walls, no water or air leaks). | ( ) | ( ) | | |
| 2.6.2 | Stretcher beds with handrails and wheels | ( ) | ( ) | Number: | Number: |
| 2.6.3 | Fetal monitor - Tococardiograph | ( ) | ( ) | Number: | Number: |
| 2.6.4 | Oxygen intakes with humidifier | ( ) | ( ) | Number: | Number: |
| 2.6.5 | Area for intercultural childbirth care. | ( ) **↓2.7** | ( ) | Number: | Number: |
| *2.6.5.a* | Is attention facilitated in an upright position? | | | *Yes……………………..*<br>*No……………………..* | *( )*<br>*( )* |
| *2.6.5.b* | Is attention facilitated in a lying position? | | | *Yes……………………..*<br>*No……………………..* | *( )*<br>*( )* |
| *2.6.5.c* | Is attention facilitated in a seated position? | | | *Yes……………………..*<br>*No……………………..* | *( )*<br>*( )* |
| *2.6.5.d* | Other | | | *Yes……………………..*<br>*Wich one___________*<br>*No……………………..* | *( )*<br><br>*( )* |

| | | | | | |
|---|---|---|---|---|---|
| **2.7 Expulsion room** | | ( ) **↓2.8** | ( ) | Number: | |
| **The following questions apply to the total number of ejection rooms in this hospital.** | | | | | |
| 2.7.1 | Clean (no dampness, cracks, holes in ceilings and walls, no water or air leaks). | ( ) | ( ) | | |
| 2.7.2 | Ceilings with smooth, washable material and sanitary baseboard. | ( ) | ( ) | | |
| 2.7.3 | Delivery table | ( ) | ( ) | Number: | Number: |
| 2.7.4 | Delivery equipment | ( ) | ( ) | Number: | Number: |
| 2.7.5 | Oxygen and suction outlets | ( ) | ( ) | Number: | Number: |
| 2.7.6 | Anesthesia equipment | ( ) | ( ) | Number: | Number: |
| 2.7.7 | Fixed or portable emergency light | ( ) | ( ) | Number: | Number: |
| 2.7.8 | Biological-Infectious Hazardous Waste Control Area | ( ) | ( ) | Number: | Number: |

| Hospital area or zone | | No | Yes | Available Units | Working Units |
|---|---|---|---|---|---|
| **2.8 Postpartum recovery area** | | ( ) **↓2.9** | ( ) | | |
| 2.8.1 | Stretcher beds with wheels and safety handrails | ( ) | ( ) | Number: | Number: |
| 2.8.2 | Suction oxygen intakes | ( ) | ( ) | Number: | Number: |
| 2.8.3 | Pulse oximeter | ( ) | ( ) | Number: | Number: |
| 2.8.4 | Dedicated refrigerator for storing medications | ( ) | ( ) | Number: | Number: |
| **2.9 Newborn care area** | | ( ) **↓2.10** | ( ) | | |
| 2.9.1 | Radiant heat cradle | ( ) | ( ) | Number: | Number: |
| 2.9.2 | Knobs for secretion suction | ( ) | ( ) | Number: | Number: |
| 2.9.3 | Fixed or portable aspirator | ( ) | ( ) | Number: | Number: |
| 2.9.4 | Oxygen tank or oxygen source | ( ) | ( ) | Number: | Number: |
| 2.9.5 | Neonatal resuscitation equipment (bag with reservoir, preterm and term newborn masks) | ( ) | ( ) | Number: | Number: |
| 2.9.6 | Supplies for neonatal resuscitation/meconium aspiration care (orogastric tubes) | ( ) | ( ) | Number: | Number: |
| 2.9.7 | 2.5, 3, 3.5 and 4 mm straight endotracheal cannulae, no balloon available | ( ) | ( ) | Number: | Number: |
| 2.9.8 | Laryngoscope complete with straight 0 and 1 blades | ( ) | ( ) | Number: | Number: |
| 2.9.9 | Binaural stethoscope with neonatal capsule | ( ) | ( ) | Number: | Number: |
| 2.9.10 | Clock in the newborn care area | ( ) | ( ) | Number: | Number: |
| **2.10 Neonatal transition area** | | ( ) **↓2.11** | ( ) | | |
| 2.10.1 | Normal crib (bacinete) | ( ) | ( ) | Number: | Number: |
| 2.10.2 | Radiant heat crib | ( ) | ( ) | Number: | Number: |
| 2.10.3 | Fixed incubator | ( ) | ( ) | Number: | Number: |
| 2.10.4 | Neonatal ventilators | ( ) | ( ) | Number: | Number: |

| Hospital area or zone | | No | Yes | Available Units | Working Units |
|---|---|---|---|---|---|
| **2.11 Surgical room** | | ( ) ↓**2.12** | ( ) | Number: | |
| **The following questions apply to the total number of operating rooms in this hospital.** | | | | | |
| 2.11.1 | Capnograph (device used to measure the concentration of carbon dioxide-anesthesiology) | ( ) | ( ) | Number: | Number: |
| 2.11.2 | Transfer or area for the transition of users. | ( ) | ( ) | | |
| 2.11.3 | Staff dressing room with boot change and transfer to white circulation | ( ) | ( ) | | |
| 2.11.4 | White circulation corridors with sink, standing soap dispensers with soap and window to CEyE and white circulation access to surgery rooms | ( ) | ( ) | | |
| 2.11.5 | Gray and white circulation well marked out | ( ) | ( ) | | |
| 2.11.6 | Air injectors and/or air conditioning | ( ) | ( ) | Number: | Number: |
| 2.11.7 | Electrical circuit connected to emergency plant with maximum 30-second start-up. | ( ) | ( ) | | |
| 2.11.8 | Folding doors for white circulation for health personnel and folding door for patient entrance and exit through gray circulation, surgical table, zenithal lamp with cold light, kidney tables and Pasteur tables and medical gases (25 m2). | ( ) | ( ) | | |
| 2.11.9 | Anesthesia machines with vaporizers | ( ) | ( ) | Number: | Number: |
| 2.11.10 | Clean (no humidity, cracks, holes in ceilings and walls, no water or air leaks). | ( ) | ( ) | | |
| 2.11.11 | Ceilings with smooth, washable material and sanitary baseboard. | ( ) | ( ) | | |
| 2.11.12 | Oxygen and suction outlets | ( ) | ( ) | Number: | Number: |
| 2.11.13 | Anesthesia equipment | ( ) | ( ) | Number: | Number: |
| 2.11.14 | Fixed or portable emergency | ( ) | ( ) | Number: | Number: |

| | | | | | |
|---|---|---|---|---|---|
| | light | | | | |

| Hospital area or zone | No | Yes | Available Units | Working Units |
|---|---|---|---|---|

| | | | | | |
|---|---|---|---|---|---|
| | **Monitors:** | | | | |
| 2.11.16 | Heart rate with ECG tracing | (  ) | (  ) | Number: | Number: |
| 2.11.17 | Respiratory rate | (  ) | (  ) | Number: | Number: |
| 2.11.18 | Non-invasive blood pressure (BP) cuff | (  ) | (  ) | Number: | Number: |
| **Instruments for the ejection room** | | **No** | **Yes** | **Quantity** | **They work** |
| 2.11.19 | Straight scissors | (  ) | (  ) | Number: | Number: |
| 2.11.20 | Curved scissors | (  ) | (  ) | Number: | Number: |
| 2.11.21 | Ring Forceps | (  ) | (  ) | Number: | Number: |
| 2.11.22 | Rochester forceps | (  ) | (  ) | Number: | Number: |
| 2.11.23 | Needle Holders | (  ) | (  ) | Number: | Number: |
| 2.11.24 | Omphalotome | (  ) | (  ) | Number: | Number: |
| 2.11.25 | Biological-Infectious Hazardous Waste Control Area | (  ) | (  ) | | |
| 2.11.26 | Recovery area in gray area | (  ) | (  ) | | |
| 2.11.27 | Stretcher beds | (  ) | (  ) | Number: | Number: |
| 2.11.28 | Oxygen intakes | (  ) | (  ) | Number: | Number: |
| 2.11.29 | Pulse oximeter | (  ) | (  ) | Number: | Number: |
| 2.11.15 | Oximeter | (  ) | (  ) | Number: | Number: |

| **2.12 Neonatal Intensive Care Unit** | | (  ) **↓2.13** | (  ) | | |
|---|---|---|---|---|---|
| 2.12.1 | Signage | (  ) | (  ) | | |
| 2.12.2 | Cleanliness (no dampness, cracks, holes in ceilings and walls, no water or air leaks) | (  ) | (  ) | | |
| 2.12.3 | Patient transfer or transition | (  ) | (  ) | | |
| 2.12.4 | Isolation filter or controlled access for staff and visitors | (  ) | (  ) | | |
| 2.12.5 | Oxygen and suction intakes per bed | (  ) | (  ) | Number: | Number: |
| 2.12.6 | Sufficient tributary spaces between cribs and bed mobilities | (  ) | (  ) | | |
| 2.12.7 | One sink per cubicle and one per secluded cubicle | (  ) | (  ) | | |

| | | | | | |
|---|---|---|---|---|---|
| 2.12.8 | Radiant heated cribs and fixed incubators | ( ) | ( ) | Number: | Number: |
| 2.12.9 | Phototherapy lamps | ( ) | ( ) | Number: | Number: |
| 2.12.10 | Neonatal ventilators | ( ) | ( ) | Number: | Number: |
| 2.12.11 | Laminar flow hood for the preparation of medicines and parenteral solution. | ( ) | ( ) | Number: | Number: |
| 2.12.12 | Area for the control of Biological-Infectious Hazardous Waste. | ( ) | ( ) | | |
| 2.12.13 | Electrical circuit connected to emergency plant | ( ) | ( ) | | |

| | | | | | |
|---|---|---|---|---|---|
| **Monitors:** | | | | | |
| 2.12.14 | Heart rate with ECG tracing | ( ) | ( ) | Number: | Number: |
| 2.12.15 | Respiratory rate and blood pressure (BP) noninvasive | ( ) | ( ) | Number: | Number: |
| 2.12.16 | Adult and pediatric cuff for BP and pulse oximetry | ( ) | ( ) | Number: | Number: |
| **2.13 Adult Intensive Care Unit** | | ( ) **↓2.14** | ( ) | | |
| 2.13.1 | Signage | ( ) | ( ) | | |
| 2.13.2 | Cleanliness (no dampness, cracks, holes in ceilings and walls, no water or air leaks) | ( ) | ( ) | | |
| 2.13.3 | Isolation filter or controlled access for staff and visitors | ( ) | ( ) | | |
| 2.13.4 | Oxygen and suction intakes per bed | ( ) | ( ) | Number: | Number: |
| 2.13.5 | Sufficient tributary spacing between beds and stretchers | ( ) | ( ) | | |
| 2.13.6 | Fans | ( ) | ( ) | Number: | Number: |
| 2.13.7 | Humidifiers | ( ) | ( ) | Number: | Number: |
| 2.13.8 | Nebulizers | ( ) | ( ) | Number: | Number: |
| 2.13.9 | Electrocardiograph | ( ) | ( ) | Number: | Number: |
| 2.13.10 | Hand wash basin | ( ) | ( ) | Number: | Number: |
| 2.13.11 | Electrical contacts and plugs, no loose wiring | ( ) | ( ) | Number: | Number: |
| This unit has: | | **No** | **Yes** | **Available Units** | **Working Units** |

| | | | | | |
|---|---|---|---|---|---|
| 2.13.12 | Electrical circuit connected to emergency plant | ( ) | ( ) | | |
| 2.13.13 | Biological-Infectious Hazardous Waste Control Area | ( ) | ( ) | | |
| 2.13.14 | Masks | ( ) | ( ) | Number: | Number: |
| 2.13.15 | Ambu | ( ) | ( ) | Number: | Number: |
| 2.13.16 | Cannulas | ( ) | ( ) | Number: | Number: |

| Hospital area or zone | | No | Yes | Available Units | Working Units |
|---|---|---|---|---|---|
| **2.14 Central Equipment and Sterilization Plant (CEYE)** | | ( ) **↓sec. 3** | ( ) | | |
| 2.14.1 | Steam sterilization equipment | ( ) | ( ) | Number: | Number: |
| 2.14.2 | Dry heat sterilization equipment | ( ) | ( ) | Number: | Number: |
| 2.14.3 | Gas Sterilization Equipment | ( ) | ( ) | Number: | Number: |
| 2.14.4 | Plasma sterilization equipment | ( ) | ( ) | Number: | Number: |
| **Others** | | | | | |
| 2.15 | Abdominal salpingoclasia equipment | ( ) | ( ) | Number: | Number: |
| 2.16 | Healing equipment | ( ) | ( ) | Number: | Number: |
| 2.17 | Surgical clothing | ( ) | ( ) | | |
| 2.18 | Telephone or radio equipment to communicate with referral hospitals | ( ) | ( ) | Number: | Number: |
| 2.19 | Transfer incubator | ( ) | ( ) | Number: | Number: |

| **Section 2 Remarks (Specify question number and comment):** |
|---|
| |
| |
| |
| |
| |
| |
| |
| |
| |

| 3. HEALTH PERSONNEL | | | | |
|---|---|---|---|---|
| **Instructions**. Answer "Yes" or "No" by checking the number in the appropriate box, or enter the information requested. Enter the total number per guard. If you have observations on any of the questions, write them down in the comments section at the end of section 3. | | | | |
| | | | | |
| **3.1 Personnel attending vaginal birth:** | | **No** | **Yes** | **How many** |
| 3.1.1 | Medicina general | ( ) | ( ) | Number: |
| 3.1.2 | Nursing | ( ) | ( ) | Number: |
| 3.1.3 | Midwifery | ( ) | ( ) | Number: |
| 3.1.4 | Physical Interns | ( ) | ( ) | Number: |
| 3.1.5 | Obstetrics Residents | ( ) | ( ) | Number: |
| 3.1.6 | Family Medicine Residents | ( ) | ( ) | Number: |
| 3.1.7 | Nursing Interns | ( ) | ( ) | Number: |
| 3.1.8 | Medical Interns | ( ) | ( ) | Number: |
| 3.1.9 | Gynecologists | ( ) | ( ) | Number: |

| | | |
|---|---|---|
| **3.2 Personnel for stabilization and resolution of complications: hospital-level measures (obstetric hemorrhages, hysterectomies, preeclampsia and eclampsia, newborn asphyxia):** | | |
| 3.2.1 | Gynecologists | Number |
| 3.2.2 | Pediatricians | Number |
| 3.2.3 | Anesthesiologists | Number |
| 3.2.4 | Surgeons | Number |

| | | |
|---|---|---|
| **3.3 Adherence to current clinical practice guidelines and pregnancy regulations** | | |
| 3.3.1 | Are all birth attendants familiar with the clinical practice guidelines for pregnancy, childbirth and puerperium care? | *Yes……………………. ( )*<br>*No……………………. ( )* |
| 3.3.2 | Are all birth attendants familiar with NOM 007 for pregnancy, childbirth and puerperium care? | *Yes……………………. ( )*<br>*No……………………. ( )* |
| 3.3.3 | Are these guidelines or norms accessible 24 hours a day? | *Yes……………………. ( )*<br>*No……………………. ( )* |
| 3.3.4 | Are these guidelines or norms up to date? | *Yes……………………. ( )*<br>*No……………………. ( )* |
| 3.3.5 | Is there any training mechanism for staff on the guidelines or norms? | *Yes……………………. ( )*<br>*No……………………. ( )* |
| 3.3.6 | Is there an audit mechanism for this? | *Yes……………………. ( )*<br>*No……………………. ( )* |

| Instructions. Mark with an "X" in the box corresponding to the informant's perception. | | | |
|---|---|---|---|
| **3.4. Obstetric personnel** | | | |
| 3.4.1 | Do you consider that communication between obstetric staff during prenatal care is clear and understandable to clients? | Always......1<br>Sometimes......2<br>Never......3 | ( )<br>( )<br>( ) |
| 3.4.2 | Do you think that the midwifery staff respects the cultural beliefs of the clients and their families (e.g., keeping the placenta, keeping amulets, etc.)? | Always......1<br>Sometimes......2<br>Never......3 | ( )<br>( )<br>( ) |
| 3.4.3 | Do you consider that communication between obstetric staff during delivery care is clear and understandable to clients? | Always......1<br>Sometimes......2<br>Never......3 | ( )<br>( )<br>( ) |
| 3.4.4 | Do you consider that the obstetric staff always speaks to you by name and looks you in the eye during your care? | Always......1<br>Sometimes......2<br>Never......3 | ( )<br>( )<br>( ) |
| 3.4.5 | Do you feel that the midwifery staff takes into consideration the needs of the client during delivery care? | Always......1<br>Sometimes......2<br>Never......3 | ( )<br>( )<br>( ) |
| 3.4.6 | Do you consider that communication between midwifery staff during postpartum care is clear and understandable to clients? | Always......1<br>Sometimes......2<br>Never......3 | ( )<br>( )<br>( ) |
| 3.4.7 | Do you consider that communication between obstetric staff and family members is clear and understandable during or after delivery and newborn care? | Always......1<br>Sometimes......2<br>Never......3 | ( )<br>( )<br>( ) |
| 3.4.8 | Do you consider that there is violence and/or harassment between staff of different hierarchies in this hospital? | Always......1<br>Sometimes......2<br>Never......3 | ( )<br>( )<br>( ) |

| Instructions. Answer "Yes" or "No" by placing an "X" in the appropriate box or enter the number requested. If you have observations on any of the questions, write them down in the observations section at the end of section 3. It is recommended that you ask the head of the medical sub-directorate/human resources or the person in charge of the obstetrics-gynecology unit to obtain this information. | | |
|---|---|---|
| **Person who provided the information :** | | |
| | | |
| **3.5 Work incentives and salaries of midwifery personnel** | | |
| 3.5.1 | Do core personnel receive any type of work incentive? | *Yes......................... ( )*<br>*No.......................... ( )* **↓ *3.5.2*** |
| 3.5.1.1 | What type of work incentive do you receive? | |
| 3.5.2 | Do fee-based personnel receive any type of work incentive? | *Yes......................... ( )*<br>*No.......................... ( )* **↓ *3.5.3*** |
| 3.5.2.1 | What type of work incentive do you receive? | |
| 3.5.3 | Do trusted personnel receive any type of work incentive? | *Yes......................... ( )*<br>*No.......................... ( )* **↓ *3.6.1*** |
| 3.5.3.1 | What type of work incentive do you receive? | |

| **3.6 Average monthly salary of health personnel:** | | | |
|---|---|---|---|
| 3.6.1 | Staff of basic obstetrician-gynecologists | $ ________________________ | **Mexican Pesos MXN** |

| | | | |
|---|---|---|---|
| 3.6.2 | Basic medical staff | $ ___________________________________ | **Mexican Pesos MXN** |
| 3.6.3 | Basic nursing staff | $ ___________________________________ | **Mexican Pesos MXN** |
| 3.6.4 | Confidence OB/GYN personnel | $ ___________________________________ | **Mexican Pesos MXN** |
| 3.6.5 | Reliable medical personnel | $ ___________________________________ | **Mexican Pesos MXN** |
| 3.6.6 | Nursing personnel on a fee basis | $ ___________________________________ | **Mexican Pesos MXN** |
| 3.6.7 | Fee-based obstetrics and gynecology personnel | $ ___________________________________ | **Mexican Pesos MXN** |
| 3.6.8 | Honorary medical personnel | $ ___________________________________ | **Mexican Pesos MXN** |
| 3.6.9 | Fee-based nursing staff | $ ___________________________________ | **Mexican Pesos MXN** |
| **Remarks from section 3 (Specify question number and comment):** | | | |
| | | | |
| | | | |
| | | | |
| | | | |
| | | | |
| | | | |
| | | | |

| | | |
|---|---|---|
| **4. SUPPLIES AND MEDICATIONS** | | |
| Instructions: For the following questions, mark with an X “Yes” or “No” whether the toco-surgery unit has the equipment or supplies indicated. If the answer is “Yes”, write the number available and the number of units operating when the legend “Number: _______” appears in the corresponding boxes. If you have observations on any of the questions, note them in the “Observations” section. | | |
| 4.1 | Has this unit had any problems with the supply of medicines or supplies? | *Yes……………………. ( )*<br>*No……………………. ( )* **↓ 4.6** |
| 4.1.1 | Which ones? | |
| 4.2 | Is there a supply of vaccines for women during the 2nd and 3rd trimester of pregnancy, according to the GPC Vaccination of pregnant women? | *Yes……………………. ( )*<br>*No……………………. ( )* |
| 4.3 | Is there a supply of vaccines for the newborn, according to the Universal Vaccination Schedule? | *Yes……………………. ( )*<br>*No……………………. ( )* |

| This unit has: | | No | Yes | Available Units | Working Units |
|---|---|---|---|---|---|
| 4.4 Supplements for women during pregnancy | | | | | |
| 4.4.1 | Iron | ( ) | ( ) | Number: | |
| 4.4.2 | Folic Acid | ( ) | ( ) | Number: | |
| 4.5 Tests | | | | | |
| 4.5.1 | Tests for human acquired immunodeficiency virus HIV in pregnant women | ( ) | ( ) | Number: | |
| 4.5.2 | Tests for the detection of urinary tract infection (general urine test) | ( ) | ( ) | Number: | |
| 4.5.3 | Tests for ABO and Rh blood grouping in pregnant women | ( ) | ( ) | Number: | |
| 4.6 Antibiotics | | | | | |
| 4.6.1 | Ampicillin (capsules and injection) | ( ) | ( ) | Number: | |
| 4.6.2 | Benzathine penicillin (injections) | ( ) | ( ) | Number: | |
| 4.6.3 | Procaine penicillin | ( ) | ( ) | Number: | |
| 4.6.4 | Cephalexin | ( ) | ( ) | Number: | |
| 4.6.5 | Metronidazole | ( ) | ( ) | Number: | |
| 4.6.6 | Clindamycin (injectable) | ( ) | ( ) | Number: | |
| 4.7 Antihypertensives | | | | | |
| 4.7.1 | Hydralazine | ( ) | ( ) | Number: | |
| 4.7.2 | Alfamethyldopa | ( ) | ( ) | Number: | |
| 4.7.3 | Nifedipine | ( ) | ( ) | Number: | |
| 4.8 Anticonvulsants | | | | | |
| 4.8.1 | Phenytoin sodium | ( ) | ( ) | Number: | |
| 4.8.2 | Diazepam | ( ) | ( ) | Number: | |
| 4.8.3 | Magnesium sulfate | ( ) | ( ) | Number: | |

| **4.9 Oxytocics** | | |
|---|---|---|
| **4.9.1** | ***<u>Oxytocin</u>*** | *Yes…………………….. ( )*<br>*No……………………… ( )* **↓ *4.9.2*** |
| 4.9.1.1 | How many ampules of oxytocin does tocosurgery have? | **Number:** |
| 4.9.1.2 | Do any of the ampoules have expired expiration dates? **(Check** | *Yes…………………….. ( )* |

| | | |
|---|---|---|
| | **one ampoule in the box that is in use)** | *No......................... ( )* |
| 4.9.1.3 | How many ampoules have expired? | **Number:** |
| 4.4.9.1.4 | Is oxytocin available in the delivery room? | *Yes......................... ( )*<br>*No......................... ( )* |
| 4.9.1.5 | Is oxytocin under refrigeration **(in toco-surgery**)? | *Yes......................... ( )*<br>*No......................... ( )* |
| **4.9.2** | ***Ergometrine / Ergonovine (E/E)*** | *Yes......................... ( )*<br>*No......................... ( )* **↓ *4.9.3*** |
| 4.9.2.1 | How many ampules of ergonovine do you have in tocosurgery? | **Number:** |
| 4.9.2.2 | Do any of the ampoules have expired expiration date? **(Check one ampoule in the box that is in use)** | *Yes......................... ( )*<br>*No......................... ( )* |
| 4.9.2.3 | How many ampoules have expired? | **Número:** |
| 4.9.2.4 | Is the E/E available in the delivery room? | *Yes......................... ( )*<br>*No......................... ( )* |
| 4.9.2.5 | Is the E/E under refrigeration **(in tocosurgery)?** | *Yes......................... ( )*<br>*No......................... ( )* |
| **4.9.3** | ***Misoprostol*** | *Yes......................... ( )*<br>*No......................... ( )* **↓ *4.9.4*** |
| 4.9.3 | How many misoprostol tablets does tocosurgery have? | **Number:** |
| 4.9.3.2 | Do any of the tablets have expired expiration dates**? (Verify from the box that it is in use)** | *Yes......................... ( )*<br>*No......................... ( )* |
| 4.9.3.3 | | **Number:** |
| 4.9.3.4 | How many are past their expiration date? | *Yes......................... ( )*<br>*No......................... ( )* |

| | | |
|---|---|---|
| **4.9.4** | ***Carbetocin*** | *Yes......................... ( )*<br>*No......................... ( )* |
| 4.9.4.1 | How many ampules of carbetocin does tocosurgery have? | **Number:** |
| 4.9.4.2 | Do any of the ampoules have expired expiration date? **(Check from the box that it is in use)** | *Yes......................... ( )*<br>*No......................... ( )* |

| | | |
|---|---|---|
| 4.9.4.3 | How many ampoules have expired? | **Number:** |
| 4.9.4.4 | Is carbetocin available in the delivery room? | *Yes…………………….. ( )*<br>*No……………………… ( )* |
| 4.9.4.5 | Is carbetocin under refrigeration **(in tocosurgery)**? | *Yes…………………….. ( )*<br>*No……………………… ( )* |
| **4.10** | **Intravenous Solutions** | |
| 4.10.1 | Sodium chloride/saline solution | *Yes…………………….. ( )*<br>*No……………………… ( )* |
| 4.10.2 | Glucose with sodium chloride | *Yes…………………….. ( )*<br>*No……………………… ( )* |
| 4.10.3 | Simple glucose solution | *Yes…………………….. ( )*<br>*No……………………… ( )* |
| 4.10.4 | Hartmann | *Yes…………………….. ( )*<br>*No……………………… ( )* |

| **This unit has:** | | **No** | **Yes** | **Available Units** | **Working Units** |
|---|---|---|---|---|---|
| **4.11** | **Prophylactic drugs in newborns** | | | **Total # of ampoules/ointments or ointments in the operating room.** | |
| 4.11.1 | Vitamin A | ( ) | ( ) | **Numer:** | |
| 4.11.2 | Vitamin K | ( ) | ( ) | **Numer:** | |
| 4.11.3 | Chloramphenicol | ( ) | ( ) | **Numer:** | |
| **Supplies for postpartum and newborn care** | | | | | |
| 4.12 | Equipment for infusion of intravenous solutions in the hospital unit. | ( ) | ( ) | **Number:** | |
| 4.13 | Do you have all of the following supplies (sanitary napkins, diapers, postpartum care clothing)? | ( ) | ( ) | | |
| 4.14 | Do you have all of the following supplies (gauze, syringes, needles, catheters, diapers, fields, clothing for newborn care)? | ( ) | ( ) | | |
| 4.15 | Supplies for umbilical cord ligation (umbilical tape, ligature or equivalent) | ( ) | ( ) | | |
| 4.16 | Vaccine application equipment (syringes, needles, primers, etc.) | ( ) | ( ) | | |
| 4.17 | 3.5 F and 5 F umbilical catheter, available at the newborn care area | ( ) | ( ) | | |

| | | | | | |
|---|---|---|---|---|---|
| 4.18 | Do you have a neonatal screening test? | (    ) | (    ) | **Number:** | |
| 4.19 | Surfactant for premature newborns in the hospital unit | (    ) | (    ) | **Number:** | |
| **Remarks from section 4 (Specify question number and comment):** | | | | | |
| | | | | | |
| | | | | | |
| | | | | | |
| | | | | | |
| | | | | | |
| | | | | | |
| | | | | | |
| | | | | | |
| | | | | | |

| **5. Use of Services** | | |
|---|---|---|
| **Statistical and epidemiological information** | | **Number** |
| 5.1 | **Total number of women registered in the CENSUS of pregnant women in the last 6 months. Specify by months overdue** (information can be found in the passbooks). | |
| 5.2 | **Total, of women first prenatal visit in the last 6 months. Specify by months overdue** (information can be found in the passbooks). | |
| 5.3 | **How many deliveries have been attended in the last six months?** | |
| 5.3.1 | **Ask the gynecology staff: What is the average length of stay after natural childbirth?** | |
| 5.4 | **How many cesarean sections have been performed in the last six months?** | |
| 5.4.1 | **Ask gynecology staff: What is the average length of stay post-cesarean section?** | |
| 5.5 | **How many abortions have been performed in the last six months?** | |
| 5.6 | **How many curettages have been performed in the last six months?** | |
| 5.7 | **How many MVAs have been performed in the last six months?** | |
| 5.8 | **How many cases of preeclampsia were diagnosed in the last six months?** | |
| 5.8.1 | **How many cases were referred in the last six months?** | |
| *If there were no cases in the previous 6 months **go to 5.9.*** *If a maximum of 10 cases were recorded, please review all the files where the event occurred and complete the information requested in 5.8.2-5.8.4.* | | |
| 5.8.2 | Number of reviewed files with a diagnosis of **preeclampsia** | |
| 5.8.3 | Of the files reviewed with a diagnosis of preeclampsia, how many received **Magnesium Sulfate (SO4Mg)** during treatment? | |
| 5.8.3 | Number of ampoules of (SO4Mg) administered during treatment (Total ampoules) | |
| 5.9 | How many cases of eclampsia were diagnosed and referred in the last six months? | |
| 5.9.1 | How many cases of eclampsia were referred in the last six months? | |
| *(If there were no cases in the previous 6 months**, go to 5.7**).* *If there were cases please review all the files where the event occurred and complete the information requested in* | | |

| *5.9.2-5.9.4.* | | |
|---|---|---|
| 5.9.2 | Number of **records** reviewed with a diagnosis of **eclampsia** | |
| 5.9.3 | Of the files reviewed with eclampsia diagnosis How many received Magnesium Sulfate (**SO4Mg**) during treatment? | |
| 5.9.4 | Number of grams of (SO4Mg) administered during treatment: | |
| | **File** | **Dosage (gr)** |
| | *1* | |
| | *2* | |
| | *3* | |
| | *4* | |
| | *5* | |
| | *6* | |
| | *7* | |
| | *8* | |
| | *9* | |
| | *10* | |
| 5.10 | How many cases of preeclampsia evolved into eclampsia in the last six months? | |
| 5.11 | How many cases of obstetric hemorrhage have been treated in the last six months? | |
| 5.12 | How many cases of obstetric hemorrhage have been referred to a referral hospital in the last six months? | |
| 5.13 | How many transfusions have been performed for obstetric hemorrhage in the last six months? | |
| 5.14 | How many obstetric hysterectomies have been performed in the last six months? | |
| 5.15 | *** If there is an Intensive Care Unit, answer: How many obstetric cases have been admitted to the Intensive Care Unit in the last six months? Otherwise go to question 5.16 | |
| 5.16 | How many cases of neonatal complications have been admitted to the intensive care unit in the last six months? | |
| 5.17 | Number of cases with APGAR less than 7 at 5 min. in the last six months? | |
| 5.18 | How many cases of maternal deaths have occurred in the last six months? | |

| *(If there were no cases in the previous 6 months **go to 5.19**)* | | |
|---|---|---|
| 5.18.1 | Specify the **causes** of each of the **maternal deaths** according to ICD-10 diagnosis codes. Use death certificates (e.g., miscarriage, obstetric hemorrhage, preeclampsia, and eclampsia). | |
| | ***No.*** | **Diagnosis** |
| | 1 | |
| | 2 | |
| | 3 | |
| | 4 | |
| | 5 | |

| | | |
|---|---|---|
| | 6 | |
| | 7 | |
| | 8 | |
| | 9 | |
| | *10* | |
| 5.19 | How many cases of perinatal deaths have occurred in the last six months? (**Perinatal deaths are defined as those occurring from birth to day 28)** > If there were no cases in the previous 6 months, **skip to 5.20.** | |
| 5.19.1 | Specify the causes of perinatal deaths (Deaths from birth to day 28) that have occurred during the last six months according to ICD-10 diagnosis codes. Use death certificates. Review a minimum of 10 files (if more than 10, add at the end of the questionnaire). | |
| | ***No.*** | **Diagnosis** |
| | 1 | |
| | 2 | |
| | 3 | |
| | 4 | |
| | 5 | |
| | 6 | |
| | 7 | |
| | 8 | |
| | 9 | |
| | *10* | |
| 5.20 | How many referrals for women with obstetric complications have been made in the last six months? | |
| 5.21 | How many referrals for neonatal complications have been made in the last six months? | |
| 5.22 | How many cases of stillbirths were reported in the last six months? | |

## 5.23 Strategic planning

**Instructions:** This information can be provided by management personnel or by those in charge of management and administration.

| The health unit makes an estimate of: | | **No** | **Yes** | **Specify the data** |
|---|---|---|---|---|
| *5.23.1* | *Fertility rate* | (   ) | (   ) | |
| *5.23.2* | *Beneficiary population of childbearing age* | (   ) | (   ) | |
| *5.23.3* | *Expected number of low-risk pregnancies* | (   ) | (   ) | |

| | | | | |
|---|---|---|---|---|
| *5.23.4* | *Expected number of complicated pregnancies* | ( ) | ( ) | |
| *5.23.5* | *Expected number of low-risk deliveries* | ( ) | ( ) | |
| *5.23.6* | *Expected number of complicated deliveries* | | | |
| *5.23.7* | *Expected number of newborns* | ( ) | ( ) | |
| *5.23.8* | *Women hospitalized for perinatal care* | ( ) | ( ) | |
| *5.23.9* | *Frequency of preterm deliveries* | ( ) | ( ) | |
| *5.23.10* | *Frequency of post-term deliveries* | ( ) | ( ) | |
| *5.23.11* | *Average number of days of NICU stay* | ( ) | ( ) | |
| *5.23.12* | *Rate of puerperal infections* | ( ) | ( ) | |
| 5.23.13 | *Does the health facility carry out strategic planning of supplies according to the population of childbearing age?* | | | *Yes……………………. ( )*<br>*No……………………. ( )* |
| 5.23.14 | *Is there an evaluation of the supply distribution systems within the hospital unit?* | | | *Yes……………………. ( )*<br>*No……………………. ( )* |
| 5.23.15 | *Is there an evaluation of drug distribution systems within the hospital unit?* | | | *Yes……………………. ( )*<br>*No……………………. ( )* |

| **Remarks from section 5 (Specify question number and comment):** |
|---|
| |
| |
| |
| |
| |
| |
| |
| |

**References:**


1. NORMA Oficial Mexicana NOM-007-SSA2-2016, Para la atención de la mujer durante el embarazo, parto y puerperio, y de la persona recién nacida.

Clinical Practice Guideline. Surveillance and management of labor in low-risk pregnancy. Mexico: Secretaría de Salud; December 11, 2014.

3. Berdichevsky, K., Diaz-Olavarrieta, C., McCarthy, K., and Blanc, A. 2014. “Validating Indicators of the Quality of Maternal Health Care: Final Report, Mexico.” Mexico City: Population Council.

4. National Institute of Public Health and Comité Promotor por una Maternidad Segura en México. Resultados 1er Taller: Calidad de la Atención en el embarazo, parto y puerperio (CAEPP). 5 November 2014.

| |
|---|
| 5. National Institute of Public Health. 2nd Workshop: Quality of Care in Pregnancy, Childbirth, Puerperium, and the Newborn (CAEPPyRN). 28 January 2016. |
| 6. Resource Model for the Planning of Medical Units of the Ministry of Health (Humanized Childbirth Unit). Dirección General de Planeación y Desarrollo en Salud (DGPLADES), Mexico 2016. |


## Collaborating institutions and participants of the CAEPPyRN Workshops:

Center for Adolescents of San Miguel de Allende, A.C. (CASA)

Center for Civic Collaboration (CCC)

Dirección General de Planeación y Desarrollo en Salud (DGPLADES) (General Directorate of Health Planning and Development)

Instituto de Seguridad y Servicios Sociales de los Trabajadores del Estado (ISSSTE) (Institute of Security and Social Services for State Workers)

National Institute of Public Health (INSP)

Secretary of Health of Morelos (SSM)

IPAS, Mexico

Promoting Committee for Safe Motherhood in Mexico (CPMS)

Mexican Institute of Social Security (IMSS)

Observatory of Maternal Mortality (OMM)

K'inal Antzetik, A.C.

National Center for Gender Equity and Reproductive Health (CNEGySR)

Directorate of Quality, Servicios de Saud, Veracruz

Center for Research and Higher Studies in Social Anthropology (CIESAS)

National Institute of Perinatology (INPer)

General Hospital of Tula, Health Services of Hidalgo (SSH)

Empowered Motherhood Collective (CME)

Independent Consultant, Grupo de Información en Reproducción Elegida, A.C. (GIRE)

Center for Maternal and Child Research of the Childbirth Study Group (CIMIGEN)

Collective for Research, Development and Education among Women, A.C. (CIDEM)

FUNDAR, Center for Analysis and Research

World Vision

Save the children

Mexican Midwifery Association (AMP)

Luna Maya, Casa de Partos

Balance A.C.

Secretary of Health of Durango (SSD)

General Hospital of Leon, Guanajuato

United Traditional Midwives Tumben Cuxtal

United Nations Population Fund, Mexico (UNFPA)

United Nations Children's Fund (UNICEF)

University of California, Sa

Center for Adolescents of San Miguel de Allende, A.C. (CASA)

Center for Civic Collaboration (CCC)
