# Supplementary material for: Developing comprehensive perinatal quality of care instruments in Mexico: An inclusive, multidisciplinary, and culturally sensitive approach
Source: PLoS One. 2026 Jul 16;21(7):e0352347. doi: 10.1371/journal.pone.0352347 (PMC13374906; doi:10.1371/journal.pone.0352347)
Supplement: S4 Appendix — (PDF) [file pone.0352347.s004.pdf]

CAEPPyRN

Proyecto: "Calidad de la Atención en el Embarazo, Parto, Puerperio y al Recién Nacido (CAEPPyRN) en México"

ANNEX 4: Semi-Structured Interview Guide for Women Users (Spanish Version)

Presentación de la entrevistadora

Gracias por su interés en participar en esta entrevista. El objetivo de la entrevista es conocer las opiniones de las mujeres que recientemente han tenido sus bebés sobre la atención durante el parto en el hospital.

Esta entrevista forma parte de una investigación realizada por el Instituto Nacional de Salud Pública (INSP). Es importante saber que las preguntas que le vamos a hacer no tienen una respuesta correcta o incorrecta, solamente queremos conocer sus opiniones para poder hacer una propuesta para mejorar la atención que se recibe en este hospital.

Antes de comenzar la entrevista, vamos a revisar una hoja que explica los derechos que tiene Usted como participante en este estudio (consentimiento informado). En caso de que esté de acuerdo, le pediremos que firme el documento. Si tiene alguna duda, me puede preguntar lo que necesite en todo momento. [Obtenga el consentimiento escrito de la participante y firme la copia del formulario para registrar la firma y la fecha.]

Número de identificación de la participante:

Fecha de la entrevista:

Fecha del nacimiento del último bebé:

Hora de inicio de la entrevista:

1. **SALUDO Y DATOS GENERALES**

   Buenos días o tardes, ¿Cómo se siente Usted hoy? Para comenzar, le quisiera preguntar, ¿cómo le gusta que la llamen?
   Felicidades por su bebé. ¿Es niño o niña? ¿Y ya tiene nombre? ¿Cómo se llama?

Vamos a comenzar a platicar sobre algunos datos generales:

1.1 ¿Me puede decir cuántos años tiene Usted? [Edad:]

1.2 ¿Cuál es su escolaridad? (Sin escolaridad [1], primaria [2], secundaria [3], media superior (escuela técnica y preparatoria) [4], universidad o superior [5], No responde)

1.3 ¿Cuál es su estado civil (Casada/Unión Libre [1], Soltera [2], Viuda/Divorciada [3], No Sabe/no está disponible [4])

1.4 ¿Cuál es su principal ocupación?

1.5 ¿Con quién vive ahora?

1.6 ¿Cuántos embarazos ha tenido? [Número:]

1.7 ¿Cuántos hijos tiene? [Número:]

1.8 ¿Alguno de sus hijos nació por cesárea? Sí [ ] No [ ]

1.9 ¿Tiene algún tipo de Seguridad Social? Sí [ ] No [ ]

Si es así, ¿cuál es? (IMSS, ISSSTE, Seguro Popular, Seguro Privado)

1.10 ¿Por qué decidió atenderse en este hospital?

1.11 ¿Cuánto tiempo tarda en llegar a este hospital desde su casa?

1.12 Antes de embarazarse, ¿fue Usted a consulta para saber cómo prepararse para su embarazo?
Sí [ ] No [ ]

2. **EXPERIENCIA DURANTE EL EMBARAZO**

Vamos a hablar un poco sobre su experiencia durante el embarazo (por ejemplo, como le fue y cómo la atendieron)

2.1 ¿Alguien le apoyó durante su embarazo? Sí [ ] No [ ] NR=no responde [ ]

2.1.1 ¿Quién?

2.1.2 ¿De qué manera?

2.2 ¿Asistió a consultas de control prenatal? Sí [ ] No [ ] NR=no responde [ ] Si no asistió, ¿cuál fue la razón? (Pase al apartado 3)

2.3 En caso afirmativo, asistió, ¿Platíqueme cómo eran sus consultas?

2.31 ¿A cuántas consultas asistió usted?

2.3.2 ¿En dónde asistió las consultas?

2.3.3 ¿Podría decirme quién la atendió durante su embarazo? (médico general, enfermera, partera, especialista, otro)

2.3.4 ¿En promedio, ¿cuánto tiempo esperó para entrar a su consulta(s)?

2.3.5 ¿Qué opina sobre la duración de la(s) consulta(s)?

2.3.6 ¿En general durante las consultas, ¿las explicaciones fueron claras y comprensibles?
Sí [ ] No [ ] NS= no sabe [ ] NR=no responde [ ]

¿Recuerda qué explicaciones le decían? Me puede dar algunos ejemplos…

¿Escucharon en algún momento sus:

¿temores? Sí [ ] No [ ] NS= no sabe [ ] NR=no responde [ ]

¿expectativas? Sí [ ] No [ ] NS= no sabe [ ] NR=no responde [ ]

¿opiniones? Sí [ ] No [ ] NS= no sabe [ ] NR=no responde [ ]

2.3.7 ¿Qué opina sobre la exploración física que recibió en la(s) consulta(s)?

2.3.8 ¿Le recetaron medicamentos o vitaminas durante su embarazo? Sí [ ] No [ ] NS= no NR=no responde [ ] (Si no, pase a2.3.9)

2.3.8.1 En caso afirmativo, ¿dónde los consiguió?

2.3.8.2 En caso afirmativo, ¿qué medicamentos o vitaminas le recetaron?

2.3.9 ¿Las consultas fueron gratuitas? Sí [ ] No [ ] NR=no responde [ ] En caso contrario, cuánto pagaba y en dónde fue que se las hicieron?

2.3.10 ¿Le solicitaron estudios de laboratorio? Sí [ ] No [ ] NR=no responde [ ] (Si no, pase a 2.3.11)

2.3.10.1 En caso afirmativo, ¿en qué lugar se los realizó?

2.3.10.2 En caso afirmativo, ¿cuál fue su costo?

2.3.11 ¿En algún momento se sintió discriminada en la atención que recibió durante las consultas?
Sí [ ] No [ ] NR=no responde [ ] Si la respuesta es afirmativa, explorar porqué razón

2.3.12 ¿Le dijeron cuáles son los signos de alarma durante su embarazo? (Si no, pase a 2.3.14)
Sí [ ] No [ ] NR=no responde [ ] ¿Me podría dar algún ejemplo?

2.3.13 ¿Qué opina sobre las explicaciones e información que le dio el personal de salud sobre:

2.3.13.1 ¿Lo que iba a pasar durante el parto?

2.3.13.2 ¿Las posibles causas para hacer una cesárea si lo necesitara y cuáles son las consecuencias?

2.3.13.3 ¿Lo que iba a pasar después de que naciera su bebé?

2.3.13.4 ¿Anticonceptivos?
2.3.13.5 ¿Cuidados personales?
2.3.13.6 ¿Lactancia materna?
2.3.13.7 ¿Alimentación?
2.3.13.8 ¿Vacunación? Para su bebé y para Usted

2.3.14 ¿Usted y su médico hicieron algún plan de parto (o plan de seguridad)? Sí [ ] No [ ] NR=no responde [ ] (Si no, pase a al apartado 3)

2.3.14.1 ¿En qué consistía?

2.3.14.2 Cuándo se fue al hospital, ¿este plan pudo seguir este plan? Sí [ ] No [ ] NR=no responde [ ] En caso contrario, me puede platicar por qué no pudo hacerlo.

3. EXPERIENCIA DURANTE EL PARTO

Ahora vamos a hablar sobre cómo le fue durante su parto

3.1 Platíqueme cómo fue el traslado para llegar aquí.

3.2 ¿Cómo supo que ya se tenía que ir al hospital? (tuvo contracciones, expulsión de moco, rompimiento de la fuente, etc.)

3.3 ¿Cómo se fue al hospital?

3.4 ¿Con quién se fue?

3.5 ¿Hubo problemas en el traslado? Sí [ ] No [ ] NR=no responde [ ] ¿Cuáles?

3.6 ¿Cuánto gastó en el traslado de ida y vuelta?

3.7 ¿La regresaron del hospital? Sí [ ] No [ ] NR=no responde [ ] (Si no, pase a 3.8)

3.7.1 En caso afirmativo, ¿por qué?

3.7.2 ¿Qué indicaciones le dieron? (que volviera cuando, y en cuanto tiempo)

3.8 ¿Cuántas veces y quiénes la revisaron antes de ingresarla al hospital?

3.9 ¿Cuánto tenía de dilatación al ingreso?

3.10 ¿Le gustó cómo la atendieron durante el ingreso? Sí [ ] No [ ] NS= no sabe NR=no responde [ ] ¿Por qué?

4. EXPERIENCIA DE PARTO DENTRO DEL HOSPITAL

Ahora vamos a platicar sobre su trabajo de parto dentro del hospital

4.1 ¿La pasaron a la sala de nacimiento? Sí [ ] No [ ] NR=no responde [ ] (Si no, pase a 4.3)

4.2 En caso afirmativo, desde que entró a la sala de parto del hospital, ¿cuántas horas estuvo ahí con otras mujeres antes de que la pasaran a la sala de nacimiento?

4.2.1 ¿Qué opina sobre ese tiempo de espera?

4.2.2 ¿En dónde estuvo esperando para ser atendida antes del parto?

4.3 ¿Cree que existieron diferencias entre el trato a las otras mujeres y el suyo? Sí [ ] No [ ] NR=no responde [ ] ¿Por qué lo piensa así?

4.4 ¿Alguien la acompañó la mayoría del tiempo durante el trabajo de parto? Sí [ ] No [ ] NR=no responde [ ] (Si no, pase a 4.5)

4.4.1 En caso afirmativo, ¿quién o quiénes fueron?

4.4.2 En caso negativo, ¿por qué?

4.5 ¿Le pidieron que se quitara la ropa frente a otras personas? Sí [ ] No [ ] NR=no responde [ ] ¿ En caso afirmativo, ¿cómo se sintió?

4.6 ¿Le explicaron de forma clara y comprensible que iba pasando y lo que le iban haciendo? Sí [ ] No [ ] NR=no responde [ ]

4.7 ¿La dejaron caminar y moverse libremente durante el trabajo de parto? Sí [ ] No [ ] NR=no responde [ ] ¿Lo hizo? Sí [ ] No [ ] NR=no responde [ ]

4.8 ¿Le ofrecieron beber algunos líquidos? Sí [ ] No [ ] NR=no responde [ ] ¿ En caso afirmativo los aceptó? Sí [ ] No [ ] NR=no responde [ ]

4.9 ¿Le realizaron el rasurado de su área púbica? Sí [ ] No [ ] NR=no responde [ ]

4.10 ¿Antes de su parto, le introdujeron por su ano un líquido para poder limpiar su intestino? Sí [ ] No [ ] NR=no responde [ ]

4.11 ¿Le pusieron suero? Sí [ ] No [ ] NS= no sabe [ ] NR=no responde [ ]

4.12 ¿Le pusieron una medicina para que naciera más rápido su bebé? Sí [ ] No [ ] NR=no responde [ ] En caso afirmativo, ¿le informaron y le explicaron por qué? Sí [ ] No [ ] NR=no responde [ ]

4.13 ¿Le pusieron anestesia en la espalda (epidural)? Sí [ ] No [ ] NS= no sabe [ ] NR=no responde [ ]

4.14 ¿Le preguntaron en qué posición quería estar Usted durante las contracciones? Sí [ ] No [ ] NR=no responde [ ] ¿En caso afirmativo, le hicieron caso? [Sí [ ] No [ ] NS= no sabe [ ] NR=no responde [ ]

4.15 ¿Le hicieron tactos vaginales? Sí [ ] No [ ] NR=no responde [ ] (Si no, pase a 4.16)

4.15.1 En caso afirmativo, ¿cuántas veces se los hicieron?

4.15.2 En caso afirmativo, ¿Le explicaron de qué se trataba? Sí [ ] No [ ] NR=no responde [ ]

4.16 ¿Escucharon el corazón de su bebé con algún aparato? [Sí [ ] No [ ] NS= no sabe [ ] NR=no responde [ ] (Si no, pase a 4.17) En caso afirmativo, ¿le explicaron para que lo hacían? [Sí [ ] No [ ] NR=no responde [ ]

4.17 ¿Le ofrecieron algún masaje durante el trabajo de parto? Sí [ ] No [ ] NR=no responde [ ] (Si no, pase a 4.18)

4.17.1 ¿Se lo hicieron? Sí [ ] No [ ] NS= no sabe [ ] NR=no responde [ ]

4.17.2 En caso afirmativo, ¿quién se lo hizo?

4.17.3 En caso afirmativo, ¿en qué parte de su cuerpo lo hizo?

4.17.4 ¿Cómo se sintió después del masaje, le sirvió?

4.18 ¿Le permitieron elegir la posición en la que usted se sentiría más cómoda para que naciera su bebé? (Si no, pase a 4.19) Sí [ ] No [ ] NR=no responde [ ]

4.18.1 ¿En qué posición fue?

4.18.2 En caso negativo, ¿Usted lo había pedido, me podría platicar más de porque no la dejaron?

4.19 ¿Apachurraron su estómago para que bajara el bebé? Sí [ ] No [ ] NR=no responde [ ]

4.20 ¿Le hicieron un corte abajo para que el bebé saliera más fácil (episiotomía)? Sí [ ] No [ ] NR=no responde [ ] ¿Le informaron que lo harían? Sí [ ] No [ ] NS= no sabe [ ] NR=no responde [ ]

4.21 Después de haber salido la placenta, ¿le introdujeron toda la mano? (Si no, pase a 3.21)
Sí [ ] No [ ] NR=no responde [ ]

4.21.1 En caso afirmativo, ¿le pusieron anestesia en la espalda? Sí [ ] No [ ] NR=no responde [ ]

4.21.2 En caso afirmativo, ¿le explicaron para qué servía esta maniobra? Sí [ ] No [ ] NR=no responde [ ]

4.22 En general durante toda la atención, ¿considera usted que la trataron con respeto?
[Sí [ ] No [ ] NR=no responde [ ]

4.23 ¿Usted considera que le dieron información durante el embarazo para que tomara decisiones durante su parto? Sí [ ] No [ ] NR=no responde [ ] ¿Me podría dar un ejemplo de esta información que le dieron? (Si no, pase a 4.25)

4.24 En el momento del parto, ¿en verdad pudo tomar algunas decisiones? Sí [ ] No [ ]
NR=no responde [ ] (Si no, pase a 3.23.2) ¿Cuáles?

4.25 ¿En los últimos momentos del parto, cuando ya iba a nacer el bebé, alguien la apoyó? Sí [ ]
No [ ] NR=no responde [ ] En caso afirmativo, ¿quién fue y de qué manera lo hizo?

4.26 ¿Le pidieron que firmara algún papel de consentimiento? Sí [ ] No [ ] NR=no responde [ ] (Si no, pase a 4.27)

4.26.1 En caso afirmativo, ¿le explicaron para qué era? Sí [ ] No [ ] NR=no responde [ ]

4.26.2 En caso afirmativo, ¿estuvo de acuerdo? Sí [ ] No [ ] NR=no responde [ ]

4.27¿Qué fue lo que más le agradó de su parto? ¿Por qué?

4.28 ¿Qué fue lo que menos le agradó? ¿Por qué?

4.29 ¿Existió algún problema durante el parto? Sí [ ] No [ ] NR=no responde [ ] Cuál fue y cómo se resolvió?

4.30 ¿El lugar de parto era frío o cálido? ¿Cómo se sintió con la temperatura?

4.31 ¿Van a pagar usted o sus familiares alguna cantidad por la atención del parto? Sí [ ] No [ ] NS= no sabe [ ] NR=no responde [ ] En caso afirmativo, ¿cuánto van a pagar?

4.32 ¿Tuvieron que hacer algún gasto en medicamentos, materiales o estudios en el parto? Sí [ ] No [ ] NS= no sabe [ ] NR=no responde [ ] En caso afirmativo, ¿cuánto gastaron, aproximadamente?

5. EXPERIENCIA EN EL POSTPARTO INMEDIATO

Ahora vamos a platicar sobre su experiencia en el posparto inmediato

5.1 ¿Se llevaron al bebé? Sí [ ] No [ ] NR=no responde [ ] En caso afirmativo, ¿le explicaron por qué y que le iban a hacer? [Sí [ ] No [ ] NR=no responde [ ]

5.2 ¿Recuerda si cortaron el cordón umbilical de inmediato después del nacimiento o si esperaron un poco?

5.3 ¿Le colocaron al bebé sobre su pecho para que pudiera tomar el pecho inmediatamente después del parto? Sí [ ] No [ ] NR=no responde [ ]

5.4 ¿Le permitieron dar pecho al bebé durante la primera hora? Sí [ ] No [ ] NR=no responde [ ]

5.5 ¿Tuvo alguna dificultad para darle de lactar en ese momento? Sí [ ] No [ ] NR=no responde [ ] En caso afirmativo, ¿cuál fue y cómo la resolvió?

5.6 ¿Le dieron algún consejo sobre cómo lactar? Sí [ ] No [ ] NR=no responde [ ] ¿En caso afirmativo, cuál fue y quién se lo dio?

5.7 ¿Qué piensa de la atención que recibió su bebé?

5.8 ¿Le dieron información sobre métodos de anticonceptivos después del parto? Sí [ ] No [ ] NR=no responde [ ]

5.9 ¿Sintió que algún personal de salud en el hospital la presionó para aceptar algún método anticonceptivo? Sí [ ] No [ ] NR=no responde [ ] ¿En caso afirmativo qué tipo de personal fue y qué método quería que aceptara?

5.9.1 ¿Se decidió por alguno? [Sí [ ] No [ ] NR=no responde [ ] ¿Cuál fue?

5.9.2 ¿Ya se lo dieron, hicieron o se lo pusieron? Sí [ ] No [ ] NR=no responde [ ]

5.10 ¿Cuánto tiempo después del nacimiento la volvieron a revisar? ¿Cómo fue la revisión?

5.11 ¿Le dieron algún tipo de medicamento después del parto? Sí [ ] No [ ] NR=no responde [ ] En caso afirmativo, ¿cuál fue?

5.12 Después de que terminó su parto, ya que estaba en recuperación, ¿alguien la apoyó? (Si no, pase a 5.13) [Sí [ ] No [ ] NR=no responde [ ]

5.12.1 ¿Quién?

5.12.2 ¿De qué manera lo hizo?

5.13 ¿Se le brindaron facilidades a sus familiares para visitarle en la hospitalización? Sí [ ] No [ ] NS= no sabe [ ] NR=no responde [ ]

5.14 Después del parto, ¿cuánto tiempo les dijeron que iba a pasar para que la dieran de alta?

5.15 ¿Le informaron a sus familiares sobre su estado de salud y el de su bebé una vez que terminó su parto? Sí [ ] No [ ] NS= no sabe [ ] NR=no responde [ ]

5.16 ¿Dejaron a su bebé todo el tiempo con usted después del parto? Sí [ ] No [ ] NR=no responde [ ]

5.17 En general, ¿tomaron en cuenta algunas de sus costumbres o consejos de sus familiares y/o conocido/as? (Tomar atole, caminar, que le guardaran la placenta...) Sí [ ] No [ ] NR=no responde [ ] (En caso negativo pase a las 5.18)

5.17.1 ¿Cuáles?

5.17.2 ¿De qué manera?

5.17.3 Si no, ¿me podría platicar más?

5.18 En total, desde que entró al hospital hasta ahora, ¿por cuánto tiempo estuvo en el hospital?

6. EXPERIENCIA EN CONSULTAS POSTNATALES (DESPUÉS DEL EGRESO)

Sólo, en caso de que la entrevista se realice durante el periodo de consultas postnatales

6.1 En promedio, ¿cuánto tiempo espera para entrar a su(s) consulta(s)?

6.1.1 ¿A cuántas consultas ha asistido hasta hoy?

6.1.2 ¿Qué opina sobre la duración de la(s) consulta(s)?

6.2 ¿Le explicaron de manera clara las ventajas de la lactancia materna? Sí [ ] No [ ] NR=no responde [ ]

6.3 ¿Le explicaron cómo darle amamantar a su bebé en alguna consulta postnatal? Sí [ ] No [ ] NR=no responde [ ]

6.4 ¿Le explicaron de manera clara en alguna de las consultas postnatales los signos de alarma durante el puerperio (42 días después del parto)? Sí [ ] No [ ] NR=no responde [ ]

6.5 ¿El personal médico respetó sus decisiones en cuanto a los anticonceptivos después del parto? Sí [ ] No [ ] NR=no responde [ ]

6.6 Si le realizaron un corte abajo durante su parto, a consecuencia de este corte, ¿ha presentado dolor al orinar o durante las relaciones sexuales? Sí [ ] No [ ] NR=no responde [ ]

6.7 ¿Las recetas de medicamentos que recibió durante las consultas estaban completas? Sí [ ] No [ ] NR=no responde [ ]

6.8 ¿Recibió apoyo durante alguna(s) de la(s) visita(s) postnatal(es)? Sí [ ] No [ ]NR=no responde [ ] En caso afirmativo de quién y para qué.

6.9 ¿Me podría decir cuánto tiempo le dedica a las consultas postnatales en promedio, desde que sale de su casa hasta que puede incorporarse a sus actividades regulares del día?

6.10 ¿Qué tipo de información y recomendaciones le han dado en las consultas después del nacimiento de su bebé para cuidar su salud?

6.10.1 ¿Cuáles han sido?

6.10.2 ¿Quién se las dio y para qué le han servido? (p.e. lactancia materna, aseo del bebé, signos de alarma en la salud del bebé, vacunación, tamizaje, etc.)

6.11 ¿En caso de haber tenido alguna complicación después del parto, el profesional realizó un seguimiento de esta durante alguna consulta postnatal? [Sí [ ] No [ ] NR=no responde [ ]] (Si no, pase a 6.12)

6.11.1 ¿Cuál fue la complicación?

6.11.2 ¿Cómo se resolvió?

6.12 Para terminar, durante su embarazo, parto, y después del parto, ¿ha sufrido algún tipo de maltrato por parte de algún familiar o del personal de salud? Sí [ ] No [ ] NR=no responde [ ] ¿Me podría explicar en qué sentido? (explorar aspectos de violencia obstétrica)

CIERRE

¿Hay algo más que le gustaría añadir a esta entrevista antes de finalizarla?

¡Muchas gracias por su participación!

**Referencias:**
1. NORMA Oficial Mexicana NOM-007-SSA2-2016, Para la atención de la mujer durante el embarazo, parto y puerperio, y de la persona recién nacida.

2. Guía de Práctica clínica. Vigilancia y manejo del trabajo de parto en embarazo de bajo riesgo. México: Secretaría de Salud; 11 de diciembre de 2014.
3. Berdichevsky, K., Diaz-Olavarrieta, C., McCarthy, K., and Blanc, A. 2014. "Validating Indicators of the Quality of Maternal Health Care: Final Report, Mexico." Mexico City: Population Council.
4. Instituto Nacional de Salud Pública y Comité Promotor por una Maternidad Segura en México. Resultados 1er Taller: Calidad de la Atención en el embarazo, parto y puerperio (CAEPP). 5 noviembre 2014.
5. Instituto Nacional de Salud Pública. 2º Taller: Calidad de la Atención en el Embarazo, Parto, Puerperio, y del Recién Nacido (CAEPPyRN). 28 enero 2016.
6. Modelo de Recursos para la Planeación de Unidades Médicas de la Secretaría de Salud (Unidad de Parto Humanizado). Dirección General de Planeación y Desarrollo en Salud (DGPLADES), México 2016.

Instituciones colaboradoras y participantes de los Talleres CAEPPyRN:

Centro para los adolescentes de San Miguel de Allende, A.C. (CASA)
Centro de Colaboración Cívica (CCC)
Dirección General de Planeación y Desarrollo en Salud (DGPLADES)
Instituto de Seguridad y Servicios Sociales de los Trabajadores del Estado (ISSSTE)
Instituto Nacional de Salud Pública (INSP)
Secretaria de Salud de Morelos (SSM)
IPAS, México
Comité Promotor por una Maternidad Segura en México (CPMS)
Instituto Mexicano del Seguro Social (IMSS)
Observatorio de Mortalidad Materna (OMM)
K'inal Antzetik, A.C.
Centro Nacional de Equidad de Género y Salud Reproductiva (CNEGySR)
Dirección de Calidad, Servicios de Saud, Veracruz
Centro de Investigaciones y Estudios Superiores en Antropología Social (CIESAS)
Instituto Nacional de Perinatología (INPer)
Hospital General de Tula, Servicios de Salud de Hidalgo (SSH)
Colectivo Maternidad Empoderada (CME)
Consultora Independiente, Grupo de Información en Reproducción Elegida, A.C. (GIRE)
Centro de Investigación Materno Infantil del Grupo de Estudios al Nacimiento (CIMIGEN)
Colectivo de Investigación, Desarrollo y Educación entre Mujeres, A.C. (CIDEM)
FUNDAR, Centro de Análisis e Investigación
World Vision
Save the children
Asociación Mexicana de Partería (AMP)
Luna Maya, Casa de Partos

Balance A.C.
Secretaria de Salud de Durango (SSD)
Hospital General de León, Guanajuato
Parteras Tradicionales Unidas Tumben Cuxtal
Fondo de Población de las Naciones Unidas, México (UNFPA)
United Nations Children's Fund (UNICEF)
Universidad de California, San Francisco (UCSF)
Partners in Health
MacArthur Foundation, México
