# Supplementary material for: Developing comprehensive perinatal quality of care instruments in Mexico: An inclusive, multidisciplinary, and culturally sensitive approach
Source: PLoS One. 2026 Jul 16;21(7):e0352347. doi: 10.1371/journal.pone.0352347 (PMC13374906; doi:10.1371/journal.pone.0352347)
Supplement: S5 Appendix — (PDF) [file pone.0352347.s005.pdf]

Proyecto: "Calidad de la Atención en el Embarazo, Parto, Puerperio y al Recién Nacido (CAEPPyRN) en México"

ANNEX 5: Childbirth Observation Instrument (Spanish Version)

Folio (número de parto observado en este lugar): [ ]

Hora de ingreso al hospital: (hh:mm) |____|____| : |____|____|

Centímetros de dilatación a la hora de ingreso (revisar expediente):_______________

Hora de inicio de la observación: (hh:mm) |____|____| : |____|____|

Centímetros de dilatación a la hora de inicio de observación (solicitar información al personal):__________________

Nombre del observador(a) del parto: ____

Fecha de la observación ________/_________/________/ dd/mm/aaaa

I. Información del Hospital
Nombre:
Clave Única de Establecimientos de Salud (CLUES):

II. Información de la Mujer

Instrucciones: La siguiente información se obtendrá por observación directa y del expediente de la mujer embarazada. En aquellas preguntas que aparezca una línea anotar lo que se pregunta, en las demás preguntas marcar con una X en la opción Si o No.

3. Fecha de nacimiento: ____/____/____/ dd/ mm /aaaa	7. Partos vaginales previos: ________ (número)
4. Número de embarazos:__________(número)	8. Cesáreas previas: ________(número)
5. Edad gestacional:__________(semanas)	9. Abortos previos: ________ (número)

6. ¿Éste es un embarazo de riesgo? (ver en expediente) SÍ: ___ No:___

6.1. ¿Cuál es el riesgo?____

IIa. Información Sociodemográfica de la Mujer.

Instrucciones: Anote en la línea el número que corresponda.

10. Máximo nivel de estudios concluido por la mujer:____________________________

1. Sin escolaridad
2. Primaria
3. Secundaria
4. Media superior (Escuela técnica y preparatoria)
5. Universidad o superior

88. No se sabe/no está disponible

11. Estado civil actual: __________________________

1. Casada / Unión libre
2. Soltera
3. Viuda / Divorciada

88. No se sabe/no está disponible

12. Número de hijas e hijos con vida actualmente: _________________(número)

13. ¿La mujer habla alguna lengua indígena? (Observar o revisar su expediente) Si: _______ No: _______

13ª. ¿Cuál lengua indígena habla la mujer? ____

14. En caso afirmativo: ¿Alguien del personal de salud se dirigió a la mujer en su lengua?
(Observar si esto ocurre antes, durante o después del parto) Si: _______ No: _______

III. Información de quien atiende el parto (quien recibe al bebé).

Instrucciones: Revisar en el expediente después de la atención del parto para recabar la información necesaria y/o preguntar al proveedor de salud. Anote la letra o marque la opción correspondiente.

15. Pregunte el rango de edad, si no responde, anote edad aparente: ( )

a) Menores de 20 años b) 20 a 24 c) 25 a 34 d) 35 a 44 e) 45 y más

16. Sexo: Mujer: ( ) Hombre: ( )

17. Número de horas continúas laboradas antes de la atención de este parto: ______________________________

18. Profesión: Escriba en el paréntesis la letra de la profesión que corresponda **( )**

a. Personal de enfermería auxiliar

b. Personal de enfermería general o técnico*

c. Personal con Licenciatura en enfermería

d. Personal de enfermería y obstetricia (LEO)

e. Personal especialista en enfermería perinatal

f. Personal de medicina de pregrado en internado

g. Personal pasante de medicina en servicio social

h. Personal de medicina general

i. Personal residente / especialista en ginecología

j. Personal residente / especialista en pediatría

k. Personal médico de otra especialidad

l. Personal de partería técnica profesional

*(*Personal de enfermería incluye estudiantes y pasantes, corroborar la profesión con el personal)*

IV. Información sobre otro personal presente durante el parto

Instrucciones: Marque con una X para señalar quién estuvo presente durante el parto (periodo de expulsión) y especifique el número de hombres y el número de mujeres para cada profesión

Profesión	**19. Presentes en el parto (X = Si)**	**19a Número de hombres**	**19b Número de mujeres**
a) Enfermera(o) General	( )	Núm:_______	Núm:_______
b) Lic. Enfermería	( )	Núm:______	Núm:_______
c) LEO (Lic. en Enfermería y Obstetricia)	( )	Núm:______	Núm:_______
d) Enfermera(o) Perinatal	( )	Núm:______	Núm:_______
e) Otras(os) (estudiantes, pasantes en enfermería)	( )	Núm:______	Núm:_______
f) Médica (o) Interno	( )	Núm:______	Núm:_______
g) Médica (o) Pasante	( )	Núm:______	Núm:_______
h) Médica (o) General	( )	Núm:______	Núm:_______
i) Residente o Especialista en G.O	( )	Núm:______	Núm:_______
j) Residente o Especialista en Pediatría	( )	Núm:______	Núm:_______
k) Médica (o) con otra especialidad	( )	Núm:______	Núm:_______
l) Otros (estudiantes, pasantes de medicina)	( )	Núm:______	Núm:_______
m) Personal de Partería Profesional	( )	Núm:______	Núm:_______
n) Otro (especificar):_______________________	( )	Núm:______	Núm:_______

**Acompañamiento de un familiar durante el parto**
o. Algún miembro de su familia acompaña a la mujer **Si: _______ No: _______**
p. En caso afirmativo, especifique ¿Cuál o cuáles miembros de la familia? _______________________________________

V. Periodo de dilatación - Primer periodo del trabajo de parto

**Instrucciones: Marque con una X para señalar si, no o no aplica (NA)**	**SI**	**NO**	**NA**
20. ¿Se brindó privacidad a la mujer (bata y/o cortinas y/o puertas)? Tiene que estar presente uno de los tres elementos.			
21. ¿Le permitieron a la mujer caminar o sentarse (fuera de la cama) durante el trabajo de parto?			
21a. ¿La mujer lo solicitó al personal?			
22. ¿Le permitieron a la mujer el consumo de líquidos vía oral durante el trabajo de parto?			
22a. ¿La mujer lo solicitó al personal?			
23. La mujer ¿Está canalizada?			
24. ¿Le administraron algún medicamento?			
		**pase p.26↓**	**pase p.26↓**

24a. Marque con una X todos los medicamentos que le administraron

a. Antibiótico	( )
b. Analgésico	( )
c. Anticonvulsivo	( )
d. Antihipertensivo	( )
e. Sulfato de Magnesio	( )
f. Corticoide para maduración pulmonar	( )
g. Otro ¿Cuál?	

**Instrucciones: Marque con una X para señalar si, no o no aplica (NA)**	**SI**	**NO**	**NA**
25. ¿Se aplicó anestesia epidural durante el trabajo de parto?			
26. ¿Se promocionó y/o facilitó la atención no farmacológica del dolor de parto? (como la deambulación, cambiar de posición, masajes, relajación, respiración y etc.) combinación de alguna de estas actividades?			
		**pase p.27↓**	**pase p.27↓**
26a. Si sí, ¿Cuál?			
27. ¿Se aplican compresas calientes y/o masaje perineal? (Cuidados para la protección del periné)			
28. ¿Le administraron oxitocina u otro uterotónico para la inducto-conducción del trabajo de parto?			
28a. Especifique uterotónico y dosis acumulada para la inducto-conducción hasta el momento de la observación (revise expediente**) uterotónico:__________dosis:_________**		**pase p.29↓**	**pase p.29↓**
29. ¿Le realizaron dilatación manual cervical?			
30. ¿Le realizaron maniobras de ampliación manual del periné?			
31. ¿Se utiliza partograma durante el trabajo de parto?			
32. ¿Le realizaron amniotomía?			

Instrucciones: Marque con una X para señalar si, no o no aplica (NA)	SI	NO	NA
33. ¿El personal de salud mencionó la existencia de un progreso anormal del trabajo de parto (sin modificaciones cervicales en 2 horas)?			
34. ¿El personal de salud mencionó la existencia de sospecha de afección del bienestar fetal o sufrimiento fetal?			
35. ¿Se realizó auscultación intermitente de la frecuencia cardiaca fetal durante el trabajo de parto (con un estetoscopio de Pinard o Doppler)?<br><br>Si sí, anote la hora en que se realiza cada auscultación			

NO: pase p.42↓ NA: pase p.42↓

(hh:mm) |____|____| : |____|____|

(hh:mm) |____|____| : |____|____|

(hh:mm) |____|____| : |____|____|

(hh:mm) |____|____| : |____|____|

(hh:mm) |____|____| : |____|____|

(hh:mm) |____|____| : |____|____|

	SI	NO	NA
42. ¿Se realiza monitoreo electrónico continuo para vigilar la frecuencia cardiaca fetal?			
43. Número de tactos vaginales que le realizan a la mujer durante el trabajo de parto:	Número:		
44. ¿Cuántas personas?:	Número:		
45. ¿Le preguntaron a la mujer si quería colocarse en alguna posición especial para tener a su bebé (sentada, parada, arrodillada, semiacostada y/o decúbito lateral, parto vertical funcional)?			

NO: pase p.47↓ NA: pase p.47↓

46 Si sí, marque cuál o cuáles fueron

a. Sentada	( )
b. Parada	( )
c. Arrodillada	( )
d. Semiacostada y/o decúbito lateral	( )
e. Parto vertical funcional	( )
f. Otra. ¿Cuál? ________________________________	( )

VI. Periodo de expulsión - Segundo periodo del trabajo de parto

47. Hora de inicio de la observación del parto: (hh:mm) |____|____| : |____|____|
(hora de ingreso a la sala de expulsión)

Instrucciones: Marque con una X para señalar si, no o no aplica (NA)	SI	NO	NA
48 ¿Si se le realizó la Maniobra de Kristeller (Presión del fondo del útero)?			
49. ¿Se realizó episiotomía?			

VII. Periodo de alumbramiento- Tercer periodo del trabajo de parto

51. Hora del nacimiento del bebé: (hh:mm) |____|____| : |____|____|

52. Hora de aplicación de oxitocina: (hh:mm) |____|____| : |____|____|

	SI	NO	NA
53. ¿Administran oxitocina después del nacimiento del bebé, pero antes de la salida de la placenta?			
53b. ¿Qué dosis de oxitocina aplicaron? Dosis:______________ (en unidades, UI)		pase p.54↓	pase p.54↓
54. ¿Le entregaron el bebé a la mamá justo después del nacimiento?			
55. ¿Existió contacto inmediato piel a piel entre la madre y el recién nacido(a)?			
55a. ¿Cuánto tiempo tuvieron contacto piel a piel? Minutos:______________		pase p.56↓	pase p.56↓

56. Hora del pinzamiento del cordón umbilical: (hh:mm) |____|____| : |____|____|

57. ¿En qué momento se realizó el pinzamiento del cordón umbilical? ( )
(seleccione sólo 1 opción y coloque la letra en el paréntesis)
- a) Inmediatamente después del nacimiento del bebé
- b) Dentro del 1er minuto después del nacimiento del bebé (entre los 11 segundos y 1 minuto de nacido)
- c) Después de 1 minuto del nacimiento del bebé

Instrucciones: Marque con una X para señalar si, no o no aplica (NA)	SI	NO	NA
58. ¿Se extrajo la placenta mediante tracción controlada o suave del cordón umbilical?			
59. ¿Se realizó contra-tracción del cordón umbilical?			

60. Hora del alumbramiento de la placenta: (hh:mm) |____|____| : |____|____|

	SI	NO	NA
61. ¿Realizaron masaje uterino (movimientos suaves de compresión en forma repetitiva con una mano en la parte inferior del abdomen de la mujer para estimular el útero)?			
		pase p.62↓	pase p.62↓

61a. ¿En qué momento realizaron el masaje uterino? ( )
(seleccione sólo 1 opción y coloque la letra en el paréntesis)
- a) Antes de la expulsión de la placenta
- b) Después de la expulsión de la placenta
- c) Antes y después de la expulsión de la placenta

Instrucciones: Marque con una X para señalar si, no o no aplica (NA)	SI	NO	NA
62. ¿La placenta y sus membranas fueron revisadas después del nacimiento del bebé?			
		pase p.64↓	pase p.64↓
63. ¿La placenta y sus membranas estaban integras y normales?			
64. ¿Realizan revisión de cavidad uterina manualmente?			
		pase p.66↓	pase p.66↓
65. ¿Realizan revisión de cavidad uterina instrumentada?			
¿Se le administró anestesia / antibiótico/ analgesia específicamente paa.ra la revisión de cavidad uterina?<br>¿Qué medicamento? _________________________ ¿Qué dosis? ________________<br>¿Qué medicamento? _________________________ ¿Qué dosis? ________________<br>¿Qué medicamento? _________________________ ¿Qué dosis? ________________		pase p.66↓	pase p.66↓
66. ¿El parto se complicó?			
66a. ¿ Qué le ocurrió?		pase p.67↓	pase p.67↓
**VIII. Después de nacimiento**			
67. ¿Secaron y estimularon al bebé dentro de los 30 segundos después del nacimiento?			
68. ¿Se tomaron signos vitales al bebé?			
68a. ¿Cuántos minutos después del nacimiento?		pase p.69↓	pase p.69↓
69. ¿Se administró Vitamina K al bebé?			
70. ¿Se aplicó antibiótico oftálmico al bebé?			
71. Hora final de la observación del parto: (hh:mm) |____|____| : |____|____| (hora egreso de la sala de expulsión)			
72. ¿Se observa lactancia materna dentro de los 60 minutos post-parto?			
73. ¿Se tomaron signos vitales a la madre?			
73a. ¿Cuántos minutos después del nacimiento?		pase p.74↓	pase p.74↓
**IX. Generales**			
74. Antes de realizar cualquier procedimiento durante el parto, ¿Se le explicó a la mujer, de forma clara y comprensible, en qué consistía lo qué se le iba a realizar?			
75. ¿La mujer firmó una carta consentimiento para procedimientos en general?			
76. Hora final de la observación: (hh:mm) |____|____| : |____|____|			

**Observaciones.**
**X. Anote las observaciones que crea convenientes sobre el parto observado. Si dejó alguna pregunta en blanco, o seleccionó la opción no aplica (NA), favor de anotar el número de la pregunta y explicar el por qué (por ejemplo: "se revisó expediente y no se encontró la respuesta o no se logró observar la acción porque la mujer llegó en periodo expulsivo").**

**Referencias**
1. NORMA Oficial Mexicana NOM-007-SSA2-2016, Para la atención de la mujer durante el embarazo, parto y puerperio, y de la persona recién nacida.
2. Guía de Práctica clínica. Vigilancia y manejo del trabajo de parto en embarazo de bajo riesgo. México: Secretaría de Salud; 11 de diciembre de 2014.
3. Berdichevsky, K., Diaz-Olavarrieta, C., McCarthy, K., and Blanc, A. 2014. "Validating Indicators of the Quality of Maternal Health Care: Final Report, Mexico." Mexico City: Population Council.
4. Instituto Nacional de Salud Pública y Comité Promotor por una Maternidad Segura en México. Resultados 1er Taller: Calidad de la Atención en el embarazo, parto y puerperio (CAEPP). 5 noviembre 2014.
5. Instituto Nacional de Salud Pública. 2º Taller: Calidad de la Atención en el Embarazo, Parto, Puerperio, y del Recién Nacido (CAEPPyRN). 28 enero 2016.
6. Modelo de Recursos para la Planeación de Unidades Médicas de la Secretaría de Salud (Unidad de Parto Humanizado). Dirección General de Planeación y Desarrollo en Salud (DGPLADES), México 2016.

Instituciones colaboradoras y participantes de los Talleres CAEPPyRN:

Centro para los adolescentes de San Miguel de Allende, A.C. (CASA)
Centro de Colaboración Cívica (CCC)
Dirección General de Planeación y Desarrollo en Salud (DGPLADES)
Instituto de Seguridad y Servicios Sociales de los Trabajadores del Estado (ISSSTE)
Instituto Nacional de Salud Pública (INSP)
Secretaria de Salud de Morelos (SSM)
IPAS, México
Comité Promotor por una Maternidad Segura en México (CPMS)
Instituto Mexicano del Seguro Social (IMSS)
Observatorio de Mortalidad Materna (OMM)

K'inal Antzetik, A.C.
Centro Nacional de Equidad de Género y Salud Reproductiva (CNEGySR)
Dirección de Calidad, Servicios de Saud, Veracruz
Centro de Investigaciones y Estudios Superiores en Antropología Social (CIESAS)
Instituto Nacional de Perinatología (INPer)
Hospital General de Tula, Servicios de Salud de Hidalgo (SSH)
Colectivo Maternidad Empoderada (CME)
Consultora Independiente, Grupo de Información en Reproducción Elegida, A.C. (GIRE)
Centro de Investigación Materno Infantil del Grupo de Estudios al Nacimiento (CIMIGEN)
Colectivo de Investigación, Desarrollo y Educación entre Mujeres, A.C. (CIDEM)
FUNDAR, Centro de Análisis e Investigación
World Vision
Save the children
Asociación Mexicana de Partería (AMP)
Luna Maya, Casa de Partos
Balance A.C.
Secretaria de Salud de Durango (SSD)
Hospital General de León, Guanajuato
Parteras Tradicionales Unidas Tumben Cuxtal
Fondo de Población de las Naciones Unidas, México (UNFPA)
United Nations Children's Fund (UNICEF)
Universidad de California, San Francisco (UCSF)
Partners in Health
MacArthur Foundation, México
