# Supplementary material for: Developing comprehensive perinatal quality of care instruments in Mexico: An inclusive, multidisciplinary, and culturally sensitive approach
Source: PLoS One. 2026 Jul 16;21(7):e0352347. doi: 10.1371/journal.pone.0352347 (PMC13374906; doi:10.1371/journal.pone.0352347)
Supplement: S6 Appendix — (PDF) [file pone.0352347.s006.pdf]

CAEPPyRN

Proyecto: "Calidad de la Atención en el Embarazo, Parto, Puerperio y al Recién Nacido (CAEPPyRN) en México"

ANNEX 6: Hospital Information Instrument (Spanish Version)

Fecha de Aplicación: _______/_______/_________ (dd/mm/aaaa)
Día Mes Año

Nombre de la persona que llena el cuestionario: ____
Nombre Apellidos

**Instrucciones: La información se obtendrá a través de observación en el sitio, revisión de libretas de labor, de sala de expulsión, de referencias y de entrevista con responsables de la unidad en el área médica (responsables de la dirección, subdirección y de las jefaturas de enfermería, enseñanza y epidemiología). Si existe información adicional relevante para el propósito de este trabajo, es posible realizar notas en los espacios en blanco de cada pregunta.**

**1. INFORMACIÓN GENERAL**	
1.1	Nombre de la institución:
1.2	CLUES (Clave Única de Establecimientos de Salud):
	d) Delegación/Municipio e) Estado f) Código Postal

a. Nombre y cargo de la(s) persona(s) que brinda(n) la información:			
a1			
	Nombre	Cargo	Apellidos
a2			
	Nombre	Cargo	Apellidos
a3			
	Nombre	Cargo	Apellidos
a4			
	Nombre	Cargo	Apellidos
a5			
	Nombre	Cargo	Apellidos

b. Especifique las libreta(s) o documento(s) de donde se obtuvo la información:	
b1	
b2	
b3	
b4	
b5	

Instrucciones: Revisar en libretas de tococirugía o estadística la siguiente información.		
1.3	Nombre del principal hospital de referencia:	
1.4	¿Cuenta con el nombre y teléfono de la persona encargada de **gestoría para los traslados**?	Si………………… ( )<br>No………………. ( ) ↓ **1.6**
1.4a	**Si es afirmativo, anotar los datos**	
	**Nombre:**	
1.4b	**Teléfono:**	
1.5	Tiempo que se tarda en llegar al hospital de referencia en vehículo:	**Minutos:**
1.6	¿Cuántas **ambulancias** funcionales y con gasolina están disponibles las 24 hrs?	**Número:**
1.7	¿Hay almenos 1 chofer disponible para la ambulancia las 24 hrs?	**Número:**
1.8	En caso de una referencia a otra unidad de salud, ¿El personal de este hospital brinda o consigue transporte para la referencia?	Si………………… ( )<br>No………………. ( )↓ ***Sec. 2***
1.9	En caso de una referencia ¿El personal de esta unidad se comunica con el personal del hospital para informarle sobre la referencia?	Si………………… ( )<br>No………………. ( )
1.10	En caso de una referencia ¿El personal de salud acompaña a la mujer?	Si………………… ( )<br>No………………. ( )

Observaciones de la sección 1 (Especifique el número de pregunta y el comentario)

2. INFRAESTRUCTURA Y OTROS RECURSOS					
**Instrucciones:** En las siguientes preguntas marque con una X "Si" o "No" si la unidad de salud dispone físicamente y en este momento del equipo, insumo o infraestructura señalado. Sí la respuesta es "Si", anote el número disponible y el número de unidades que funcionan cuando aparezca en las casillas correspondientes la leyenda "Número:_______". Si tiene observaciones en alguna de las preguntas, anótelas en la sección de "Observaciones".					
**Área o zona hospitalaria**		**No**	**Si**	**Unidades Disponibles**	**Unidades que Funcionan**
**2.1 Servicio de laboratorio**		( )	( )		
**2.2 Servicio de imagenología obstétrica**		( )	( )		
**2.3 Servicio de ultrasonografía obstétrica**		( )	( )		
**2.4 Sala de espera de admisión obstétrica**		( ) **↓2.5**	( )		
2.4.1	Señalizaciones en la lengua de la región	( )	( )		
2.4.2	Rampas para el acceso de usuarias	( )	( )	Número:	Número:
2.4.3	Iluminación apropiada y completa	( )	( )		
2.4.4	Ruta de evacuación y extintores colocados en el lugar apropiado(de acuerdo a la NOM-002-STPS-2000)	( )	( )		
2.4.5	Aire acondicionado	( )	( )	Número:	Número:
2.4.6	Zona para realizar triage obstétrico	( )	( )		
2.4.7	Los espacios están en buenas condiciones? (Por ejemplo: paredes sin humedad, cuarteaduras, orificios, fugas de agua, goteras)	( )	( )		
2.4.8	Baño para hombres	( ) **↓2.4.9**	( )	Número:	Número:
2.4.8.1	*¿Los baños para hombres se pueden utilizar por parte de pacientes y/o acompañantes?*			*Si..........................* ( )<br>*No.........................* ( )	
2.4.8.2				*Si..........................* ( )	

	¿Los baños para hombres cuentan con espacios físicos para su acceso y uso por personas con discapacidad?			*No......................... ( )*	
2.4.8.3	*¿Los baños para hombres cuentan con cambiadores de pañal?*			*Si........................... ( )*<br>*No......................... ( )*	
2.4.9	Baño para mujeres	( ) **↓2.5**	( )	Número:	Número:
2.4.9.1	*¿Los baños para mujeres se pueden utilizar por parte de pacientes y/o acompañantes?*			*Si........................... ( )*<br>*No......................... ( )*	
2.4.9.2	*¿Los baños para mujeres cuentan con espacios físicos para su acceso y uso por personas con discapacidad?*			*Si........................... ( )*<br>*No......................... ( )*	
2.4.9.3	*¿Los baños para mujeres cuentan con cambiadores de pañal?*			*Si........................... ( )*<br>*No......................... ( )*	

**Área o zona hospitalaria**		**No**	**Si**	**Unidades Disponibles**	**Unidades que Funcionan**
**2.5 Módulo MATER / Urgencias**		( ) **↓2.6**	( )		
2.5.1	Iluminación y ventilación adecuadas	( )	( )		
2.5.2	Espacio para que se realice el lavado de manos (lavabo)	( )	( )	Número:	Número:
2.5.3	Silla para la mujer embarazada	( )	( )	Número:	Número:
2.5.4	Lámpara de chicote	( )	( )	Número:	Número:
2.5.5	Negatoscopio	( )	( )	Número:	Número:
2.5.6	Mesa de exploración con pierneras	( )	( )	Número:	Número:
2.5.7	Biombos o cortinas para ofrecer privacidad a la usuaria	( )	( )	Número:	Número:
2.5.8	Esfigmomanómetro	( )	( )	Número:	Número:
2.5.9	Estetoscopio biauricular	( )	( )	Número:	Número:
2.5.10	Estuche de diagnóstico completo	( )	( )	Número:	Número:
2.5.11	Báscula	( )	( )	Número:	Número:
2.5.12	Estadímetro	( )	( )	Número:	Número:
2.5.13	Termómetro	( )	( )	Número:	Número:
2.5.14	Espejo vaginal desechable	( )	( )	Número:	Número:
2.5.15	Fonodetector portátil de latidos fetales (Doppler)	( )	( )	Número:	Número:

Área o zona hospitalaria		No	Si	Unidades Disponibles	Unidades que Funcionan
**2.6 Sala de labor**		( ) **↓2.7**	( )	Número:	
**Las siguientes preguntas aplican para el total de salas de labor en este hospital.**					
2.6.1	Limpia (sin humedad, cuarteaduras, orificios en plafones y paredes, ni fugas de agua o aire)	( )	( )		
2.6.2	Camas-camillas con barandal y ruedas	( )	( )	Número:	Número:
2.6.3	Monitor Fetal - Tococardiógrafo	( )	( )	Número:	Número:
2.6.4	Tomas de oxígeno con humidificador	( )	( )	Número:	Número:
2.6.5	Área para la atención de partos de forma intercultural	( ) **↓2.7**	( )	Número:	Número:
2.6.5.a	*¿Se facilita la atención en posición vertical?*			*Si……………………..*<br>*No…………………….*	*( )*<br>*( )*
2.6.5.b	*¿Se facilita la atención en posición acostada?*			*Si……………………..*<br>*No…………………….*	*( )*<br>*( )*
2.6.5.c	*¿Se facilita la atención en posición sentada?*			*Si……………………..*<br>*No…………………….*	*( )*<br>*( )*
2.6.5.d	*Otra*			*Si……………………..*<br>*¿Cuál?:______________________*<br>*No…………………….*	*( )*<br><br>*( )*

**2.7 Sala de expulsión**		( ) **↓2.8**	( )	Número:	
**Las siguientes preguntas aplican para el total de salas de expulsión en este hospital.**					
2.7.1	Limpia (sin humedad, cuarteaduras, orificios en plafones y paredes, ni fugas de agua o aire)	( )	( )		
2.7.2	Techos con material liso y lavable, y zoclo sanitario	( )	( )		
2.7.3	Mesa para la atención del parto	( )	( )	Número:	Número:
2.7.4	Equipo de parto	( )	( )	Número:	
2.7.5	Tomas de oxígeno y succión	( )	( )	Número:	Número:
2.7.6	Equipo de anestesia	( )	( )	Número:	Número:
2.7.7	Lámpara para emergencia fija o portátil	( )	( )	Número:	Número:
2.7.8	Zona para el control de los Residuos Peligrosos Biológico-Infecciosos	( )	( )	Número:	Número:

Área o zona hospitalaria		No	Si	Unidades Disponibles	Unidades que Funcionan
**2.8 Área de recuperación postparto**		( ) **↓2.9**	( )		
2.8.1	Camas-camillas con ruedas y barandales de seguridad	( )	( )	Número:	Número:
2.8.2	Tomas de oxígeno de succión	( )	( )	Número:	Número:
2.8.3	Oxímetro de pulso	( )	( )	Número:	Número:
2.8.4	Refrigerador exclusivo para guardar medicamentos	( )	( )	Número:	Número:
**2.9 Área de atención de recién nacidos**		( ) **↓2.10**	( )		
2.9.1	Cuna de calor radiante	( )	( )	Número:	Número:
2.9.2	Perillas para succión de secreciones	( )	( )	Número:	Número:
2.9.3	Aspirador fijo o portátil	( )	( )	Número:	Número:
2.9.4	Tanque o toma de oxígeno	( )	( )	Número:	Número:
2.9.5	Equipo para la reanimación neonatal (bolsa con reservorio, mascarillas para prematuro y recién nacido a término)	( )	( )	Número:	Número:
2.9.6	Insumos para la reanimación neonatal/atención por aspiración de meconio (sondas orogástricas)	( )	( )	Número:	Número:
2.9.7	Cánulas endotraqueales de 2.5, 3, 3.5 y 4 mm rectas, sin globo disponibles	( )	( )	Número:	Número:
2.9.8	Laringoscopio completo con hojas rectas del 0 y 1	( )	( )	Número:	Número:
2.9.9	Estetoscopio biauricular con capsula neonatal	( )	( )	Número:	Número:
2.9.10	Reloj en el área de atención al recién nacido	( )	( )	Número:	Número:
**2.10 Área de transición neonatal**		( ) **↓2.11**	( )		
2.10.1	Cuna normal (bacinete)	( )	( )	Número:	Número:
2.10.2	Cuna de calor radiante	( )	( )	Número:	Número:
2.10.3	Incubadora fija	( )	( )	Número:	Número:
2.10.4	Ventiladores neonatales	( )	( )	Número:	Número:

Área o zona hospitalaria		No	Si	Unidades Disponibles	Unidades que Funcionan
**2.11 Sala quirúrgica**		( )<br>↓**2.12**	( )	Número:	
**Las siguientes preguntas aplican para el total de salas quirúrgicas en este hospital.**					
2.11.1	Capnógrafo (Aparato utilizado para medir la concentración de dióxido de carbono–anestesiología)	( )	( )	Número:	Número:
2.11.2	Transfer o zona para la transición de usuarias	( )	( )		
2.11.3	Vestidor de personal con cambio de botas y transferencia hacia la circulación blanca	( )	( )		
2.11.4	Pasillos de circulación blanca con lavabo, jaboneras de pie con jabón y ventana a CEyE y acceso por circulación blanca a las salas de cirugía	( )	( )		
2.11.5	Circulación gris y blanca bien delimitadas	( )	( )		
2.11.6	Inyectores de aire y/o aire acondicionado	( )	( )	Número:	Número:
2.11.7	Circuito eléctrico conectado a planta de emergencia con arranque máximo de 30 segundos	( )	( )		
2.11.8	Puertas abatibles por circulación blanca para el personal de salud y puerta abatible para entrada y salida del paciente por circulación gris, mesa quirúrgica, lámpara cenital con luz fría, mesas de riñón y mesas de Pasteur y gases medicinales (25 m2)	( )	( )		
2.11.9	Máquinas de anestesia con vaporizadores	( )	( )	Número:	Número:
2.11.10	Limpia (sin humedad, cuarteaduras, orificios en plafones y paredes, ni fugas de agua o aire)	( )	( )		
2.11.11	Techos con material liso y lavable, y zoclo sanitario	( )	( )		
2.11.12	Tomas de oxígeno y succión	( )	( )	Número:	Número:
2.11.13	Equipo de anestesia	( )	( )	Número:	Número:
2.11.14	Lámpara para emergencia fija o portátil	( )	( )	Número:	Número:

Área o zona hospitalaria	No	Si	Unidades Disponibles	Unidades que Funcionan

	**Monitores:**				
2.11.16	Frecuencia cardiaca con trazo ECG	( )	( )	Número:	Número:
2.11.17	Frecuencia respiratoria	( )	( )	Número:	Número:
2.11.18	Brazalete de tensión arterial (TA) no invasiva	( )	( )	Número:	Número:
**Instrumental para la sala de expulsión**		**No**	**Si**	**Cantidad**	**Funcionan**
2.11.19	Tijeras rectas	( )	( )	Número:	Número:
2.11.20	Tijeras curvas	( )	( )	Número:	Número:
2.11.21	Pinza de anillos	( )	( )	Número:	Número:
2.11.22	Pinzas de Rochester	( )	( )	Número:	Número:
2.11.23	Porta agujas	( )	( )	Número:	Número:
2.11.24	Onfalotomo	( )	( )	Número:	Número:
2.11.25	Zona para el control de los Residuos Peligrosos Biológico-Infecciosos	( )	( )		
2.11.26	Área de recuperación en área gris	( )	( )		
2.11.27	Camas-camillas	( )	( )	Número:	Número:
2.11.28	Tomas de oxígeno	( )	( )	Número:	Número:
2.11.29	Oxímetro de pulso	( )	( )	Número:	Número:
2.11.15	Oxímetro	( )	( )	Número:	Número:

**2.12 Unidad de Cuidados Intensivos Neonatales**		( ) **↓2.13**	( )		
2.12.1	Señalización	( )	( )		
2.12.2	Limpieza (sin humedad, cuarteaduras, orificios en plafones y paredes, ni fugas de agua o aire)	( )	( )		
2.12.3	Transfer o transición de pacientes	( )	( )		
2.12.4	Filtro de aislamiento o acceso controlado para personal y visitantes	( )	( )		
2.12.5	Tomas de oxígeno y succión por cama	( )	( )	Número:	Número:
2.12.6	Espacios tributarios suficientes entre una cuna y otra y movilidades de camas	( )	( )		

2.12.7	Un lavabo por cubículo y uno por aislado	( )	( )		
2.12.8	Cunas de calor radiante e incubadoras fijas	( )	( )	Número:	Número:
2.12.9	Lámparas de fototerapia	( )	( )	Número:	Número:
2.12.10	Ventiladores neonatales	( )	( )	Número:	Número:
2.12.11	Campana de flujo laminar para preparación de medicamentos y solución parenteral	( )	( )	Número:	Número:
2.12.12	Zona para el control de los Residuos Peligrosos Biológico-Infecciosos	( )	( )		
2.12.13	Circuito eléctrico conectado a planta de emergencia	( )	( )		

**Monitores:**					
2.12.14	Frecuencia cardiaca con trazo ECG	( )	( )	Número:	Número:
2.12.15	Frecuencia respiratoria y tensión arteriall (TA) no invasiva	( )	( )	Número:	Número:
2.12.16	Brazalete de adulto y pediátrico para TA y oximetría de pulso	( )	( )	Número:	Número:
**2.13 Unidad de Cuidados Intensivos Adultos**		( ) **↓2.14**	( )		
2.13.1	Señalización	( )	( )		
2.13.2	Limpieza (sin humedad, cuarteaduras, orificios en plafones y paredes, ni fugas de agua o aire)	( )	( )		
2.13.3	Filtro de aislamiento o acceso controlado para personal y visitantes	( )	( )		
2.13.4	Tomas de oxígeno y succión por cama	( )	( )	Número:	Número:
2.13.5	Espacios tributarios suficientes entre una cama-camilla y otra	( )	( )		
2.13.6	Ventiladores	( )	( )	Número:	Número:
2.13.7	Humidificadores	( )	( )	Número:	Número:
2.13.8	Nebulizadores	( )	( )	Número:	Número:
2.13.9	Electrocardiógrafo	( )	( )	Número:	Número:

2.13.10	Lavamanos	( )	( )	Número:	Número:
2.13.11	Contactos eléctricos y enchufes, sin cables sueltos	( )	( )	Número:	Número:
		**No**	**Si**	**Unidades Disponibles**	**Unidades que Funcionan**
2.13.12	Circuito eléctrico conectado a planta de emergencia	( )	( )		
2.13.13	Zona para el control de los Residuos Peligrosos Biológico-Infecciosos	( )	( )		
2.13.14	Máscaras	( )	( )	Número:	Número:
2.13.15	Ambú	( )	( )	Número:	Número:
2.13.16	Cánulas	( )	( )	Número:	Número:

**Área o zona hospitalaria**		**No**	**Si**	**Unidades Disponibles**	**Unidades que Funcionan**
**2.14 Central de Equipos y Esterilización (CEYE)**		( ) ↓sec. 3	( )		
2.14.1	Equipo de esterilización vapor	( )	( )	Número:	Número:
2.14.2	Equipo de esterilización calor seco	( )	( )	Número:	Número:
2.14.3	Equipo de esterilización gas	( )	( )	Número:	Número:
2.14.4	Equipo de esterilización plasma	( )	( )	Número:	Número:
**Otros**					
2.15	Equipos de salpingoclasia abdominal	( )	( )	Número:	Número:
2.16	Equipo de curación	( )	( )	Número:	Número:
2.17	Ropa quirúrgica	( )	( )		
2.18	Teléfono o equipo de radio para comunicarse con los hospitales de referencia	( )	( )	Número:	Número:
2.19	Incubadora de traslado	( )	( )	Número:	Número:

**Observaciones de la sección 2 (Especifique el número de pregunta y el comentario):**

3. PERSONAL DE SALUD				
**Instrucciones.** Responda "Sí" o "No" marcando el número en la casilla correspondiente, o bien coloque la información que se solicita. Colocar el número total por guardia. Si tienes observaciones en alguna de las preguntas, anótelas en la sección de observaciones que viene al final de la sección 3. Se le recomienda preguntar a personal en formación.				
**3.1 Personal que atiende el parto por vía vaginal:**		**No**	**Si**	**Cuántos**
3.1.1	Medicina general	( )	( )	Número:
3.1.2	Enfermería	( )	( )	Número:
3.1.3	Partería	( )	( )	Número:
3.1.4	Médicos(as) interno(as)	( )	( )	Número:
3.1.5	Residentes de obstetricia	( )	( )	Número:
3.1.6	Residentes de medicina familiar	( )	( )	Número:
3.1.7	Pasantes de enfermería	( )	( )	Número:
3.1.8	Pasantes de medicina	( )	( )	Número:
3.1.9	Ginecólogos(as)	( )	( )	Número:

**3.2 Personal para estabilización y resolución de complicaciones: medidas a nivel hospital (hemorragias obstétricas, histerectomías, preeclampsias y eclampsias, asfixias en el recién nacido):**		
3.2.1	Ginecólogos(as)	Número:
3.2.2	Pediatras	Número:
3.2.3	Anestesiólogos(as)	Número:
3.2.4	Cirujanos(as)	Número:

**3.3 Adherencia a las guías de practica clínica y normatividad vigentes del embarazo**		
3.3.1	¿Todo el personal que atiende partos, conoce las guías de práctica clínica de la atención del embarazo, parto y puerperio?	*Si*.......................... ( )<br>*No*......................... ( )
3.3.2	¿Todo el personal que atiende partos, conoce la NOM 007 para la atención del embarazo, parto y puerperio?	*Si*.......................... ( )<br>*No*......................... ( )
3.3.3	¿Estas guías o normas están accesibles las 24 hrs del día?	*Si*.......................... ( )<br>*No*......................... ( )
3.3.4	¿Estas guías o normas están actualizadas?	*Si*.......................... ( )<br>*No*......................... ( )
3.3.5	¿Hay algún mecanismo de capacitación para el personal, sobre las guías o normas?	*Si*.......................... ( )<br>*No*......................... ( )
3.3.6	¿Existe algún mecanismo de auditoria para esto?	*Si*.......................... ( )

		No......................... ( )

**Instrucciones.** Marque con una "X" en la casilla correspondiente a la percepción del informante			
**3.4. Personal de obstetricia**			
3.4.1	¿Considera usted que la comunicación entre el personal de obstetricia durante la atención prenatal es clara y comprensible para las usuarias?	Siempre......1<br>Algunas veces......2<br>Nunca......3	( )<br>( )<br>( )
3.4.2	¿Considera usted que el personal de obstetricia respeta las creencias culturales de las usuarias y de sus familiares? (Por ejemplo: quedarse con la placenta, mantener amuletos, ¿entre otros)?	Siempre......1<br>Algunas veces......2<br>Nunca......3	( )<br>( )<br>( )
3.4.3	¿Considera usted que la comunicación entre el personal de obstetricia durante la atención del parto es clara y comprensible para las usuarias?	Siempre......1<br>Algunas veces......2<br>Nunca......3	( )<br>( )<br>( )
3.4.4	¿Considera usted que el personal de obstetricia en todo momento le habla por su nombre y mira a los ojos a las usuarias durante su atención?	Siempre......1<br>Algunas veces......2<br>Nunca......3	( )<br>( )<br>( )
3.4.5	¿Considera usted que el personal de obstetricia toma en consideración de las necesidades la atención del parto es clara y comprensible para las usuarias?	Siempre......1<br>Algunas veces......2<br>Nunca......3	( )<br>( )<br>( )
3.4.6	¿Considera usted que la comunicación entre el personal de obstetricia durante la atención del puerperio es clara y comprensible para las usuarias?	Siempre......1<br>Algunas veces......2<br>Nunca......3	( )<br>( )<br>( )
3.4.7	¿Considera usted que la comunicación entre el personal de obstetricia y los familiares es clara y entendible durante o posterior a la atención del parto y personas recién nacidas?	Siempre......1<br>Algunas veces......2<br>Nunca......3	( )<br>( )<br>( )
3.4.8	¿Considera usted que en este hospital existe violencia y/o acoso laboral entre personal de diferentes jerarquías?	Siempre......1<br>Algunas veces......2<br>Nunca......3	( )<br>( )<br>( )

**Instrucciones.** Responda "Sí" o "No" marcando con una "X" en la casilla correspondiente o coloque el número que se le solicite. Si tienes observaciones en alguna de las preguntas, anótelas en la sección de observaciones que viene al final de la sección 3. Se le recomienda preguntar al o la titular de la subdirección médica/recursos humanos o el o la responsable de la unidad de gineco-obstetricia para obtener esta información.		
**Persona que brindó la información :**		
**3.5 Incentivos laborales y salarios del personal de obstetricia**		
3.5.1	¿El personal de base recibe algún tipo de incentivo laboral?	*Si...........................* ( )<br>*No.........................* ( ) **↓ 3.5.2**
3.5.1.1	¿Cuál tipo de incentivo laboral recibe?	
3.5.2	¿El personal de honorarios recibe algún tipo de incentivo laboral?	*Si...........................* ( )<br>*No.........................* ( ) **↓ 3.5.3**
3.5.2.1	¿Cuál tipo de incentivo laboral recibe?	
3.5.3	¿El personal de confianza recibe algún tipo de incentivo laboral?	*Si...........................* ( )<br>*No.........................* ( ) **↓ 3.6.1**

3.5.3.1	¿Cuál tipo de incentivo laboral recibe?

**3.6 Salario mensual promedio personal de salud:**			
3.6.1	Personal de gineco-obstetras de base	$ ________________________________	**Pesos MXN**
3.6.2	Personal de medicina de base	$ ________________________________	**Pesos MXN**
3.6.3	Personal de enfermería de base	$ ________________________________	**Pesos MXN**
3.6.4	Personal de gineco-obstetricia de confianza	$ ________________________________	**Pesos MXN**
3.6.5	Personal de medicina de confianza	$ ________________________________	**Pesos MXN**
3.6.6	Personal de enfermería de confianza	$ ________________________________	**Pesos MXN**
3.6.7	Personal de gineco-obstetricia de honorarios	$ ________________________________	**Pesos MXN**
3.6.8	Personal de medicina de honorarios	$ ________________________________	**Pesos MXN**
3.6.9	Personal de enfermería de honorarios	$ ________________________________	**Pesos MXN**

**Observaciones de la sección 3 (Especifique el número de pregunta y el comentario):**

4. INSUMOS Y MEDICAMENTOS

Instrucciones: En las siguientes preguntas marque con una X “Si" o "No" si la unidad de tococirugía dispone del equipo o insumo señalado. Sí la respuesta es "Si", anote el número disponible y el número de unidades que funcionan cuando aparezca en las casillas correspondientes la leyenda "Número: _______". Si tiene observaciones en alguna de las preguntas, anótelas en la sección de "Observaciones".

4.1	¿Esta unidad ha tenido algunos problemas de abasto de medicamentos o insumos?	*Si.........................* ( ) *No........................* ( ) **↓ *4.6***
4.1.1	¿Cuáles?	
4.2	¿Hay abastecimiento de vacunas para la mujer durante el 2 y 3 trimestre del embarazo, según la GPC Vacunación de la embarazada?	*Si.........................* ( ) *No........................* ( )

4.3	¿Hay abastecimiento de vacunas para el recién nacido, según el Esquema de Vacunación Universal?	*Si……………………… ( )*<br>*No……………………. ( )*

Esta unidad cuenta con:		No	Si	Cantidad	Funcionan
**4.4 Suplementos para la mujer durante el embarazo**					
4.4.1	Hierro	( )	( )	Número:	
4.4.2	Ácido Fólico	( )	( )	Número:	
**4.5 Pruebas**					
4.5.1	Pruebas para la detección del virus de la inmunodeficiencia adquirida humana VIH en mujeres embarazadas	( )	( )	Número:	
4.5.2	Pruebas para la detección de infección urinaria (examen general de orina)	( )	( )	Número:	
4.5.3	Pruebas para la detección de grupo sanguíneo ABO y Rh en mujeres embarazadas	( )	( )	Número:	
**4.6 Antibióticos**					
4.6.1	Ampicilina (cápsulas e inyección)	( )	( )	Número:	
4.6.2	Penicilina Benzatínica (inyecciones)	( )	( )	Número:	
4.6.3	Penicilina procaínica	( )	( )	Número:	
4.6.4	Cefalexina	( )	( )	Número:	
4.6.5	Metronidazol	( )	( )	Número:	
4.6.6	Clindamicina (inyectable)	( )	( )	Número:	
**4.7 Antihipertensivos**					
4.7.1	Hidralazina	( )	( )	Número:	
4.7.2	Alfametildopa	( )	( )	Número:	
4.7.3	Nifedipino	( )	( )	Número:	
**4.8 Anticonvulsivos**					
4.8.1	Fenitoína sódica	( )	( )	Número:	
4.8.2	Diazepam	( )	( )	Número:	
4.8.3	Sulfato de magnesio	( )	( )	Número:	

4.9 Oxitócicos		
**4.9.1**	***Oxitocina***	*Si.......................... ( )*<br>*No......................... ( )* **↓ *4.9.2***
4.9.1.1	¿Cuántas ámpulas de oxitocina tiene tococirugía?	**Número:**
4.9.1.2	¿Algunas de las ámpulas tienen fecha de caducidad vencida?<br>**(Verificar una ámpula de la caja que esté en uso)**	*Si.......................... ( )*<br>*No......................... ( )*
4.9.1.3	¿Cuántas ámpulas tienen fecha de caducidad vencida?	**Número:**
4.4.9.1.4	¿Hay oxitocina disponible en la sala de expulsión?	*Si.......................... ( )*<br>*No......................... ( )*
4.9.1.5	¿La oxitocina se encuentra bajo refrigeración?<br>**(En tococirugía)**	*Si.......................... ( )*<br>*No......................... ( )*
**4.9.2**	***Ergometrina / Ergonovina (E/E)***	*Si.......................... ( )*<br>*No......................... ( )* **↓ *4.9.3***
4.9.2.1	¿Cuántas ámpulas de ergonovina tienen en tococirugía?	**Número:**
4.9.2.2	¿Algunas de las ámpulas tienen fecha de caducidad vencida?<br>**(Verificar una ámpula de la caja que esté en uso)**	*Si.......................... ( )*<br>*No......................... ( )*
4.9.2.3	¿Cuántas ámpulas tienen fecha de caducidad vencida?	**Número:**
4.9.2.4	¿Hay E/E disponible en la sala de expulsión?	*Si.......................... ( )*<br>*No......................... ( )*
4.9.2.5	¿La E/E se encuentra bajo refrigeración?<br>**(En tococirugía)**	*Si.......................... ( )*<br>*No......................... ( )*
**4.9.3**	***Misoprostol***	*Si.......................... ( )*<br>*No......................... ( )* **↓ *4.9.4***
4.9.3	¿Cuántas tabletas de misoprostol tiene tococirugía?	**Número:**

4.9.3.2	¿Algunas de las-tabletas tienen fecha de caducidad vencida? **(Verificar de la caja que esté en uso)**	*Si.......................... ( )*<br>*No........................ ( )*
4.9.3.3	¿Cuántas tienen fecha de caducidad vencida?	**Número:**
4.9.3.4	¿Hay misoprostol disponible en la sala de expulsión?	*Si.......................... ( )*<br>*No........................ ( )*

**4.9.4**	***<u>Carbetocina</u>***	*Si.......................... ( )*<br>*No........................ ( )*
4.9.4.1	¿Cuántas ámpulas de carbetocina tiene tococirugía?	**Número:**
4.9.4.2	¿Algunas de las ámpulas tienen fecha de caducidad vencida? **(Verificar de la caja que esté en uso)**	*Si.......................... ( )*<br>*No........................ ( )*
4.9.4.3	¿Cuántas ámpulas tienen fecha de caducidad vencida?	**Número:**
4.9.4.4	¿Hay carbetocina disponible en la sala de expulsión?	*Si.......................... ( )*<br>*No........................ ( )*
4.9.4.5	¿La carbetocina se encuentra bajo refrigeración? **(En tococirugía)**	*Si.......................... ( )*<br>*No........................ ( )*
**4.10**	**Soluciones Intravenosas**	
4.10.1	Cloruro de sodio/ solución salina	*Si.......................... ( )*<br>*No........................ ( )*
4.10.2	Glucosa con cloruro de sodio	*Si.......................... ( )*<br>*No........................ ( )*
4.10.3	Solución glucosa simple	*Si.......................... ( )*<br>*No........................ ( )*
4.10.4	Hartmann	*Si.......................... ( )*<br>*No........................ ( )*

Esta unidad cuenta con:		No	Si	Cantidad	Funcionan
**4.11**	**Medicamentos de profilaxis en el recién nacido**			**# total de ámpulas/goteros o ungüentos existentes en la sala de tococirugía.**	
4.11.1	Vitamina A	( )	( )	**Número:**	
4.11.2	Vitamina K	( )	( )	**Número:**	
4.11.3	Cloranfenicol	( )	( )	**Número:**	
**Insumos para el postparto y la atención del recien nacido**					
4.12	Equipo para la infusión de soluciones intravenosas en la unidad hospitalaria	( )	( )	**Número:**	
4.13	¿Cuenta con todos los siguientes insumos? (toallas sanitarias, pañales, ropa para vigilancia del posparto)	( )	( )		
4.14	¿Cuenta con todos los siguientes insumos? (gasas, jeringas, agujas, sondas, pañales, campos, ropa para la atención del recién nacido)	( )	( )		
4.15	Insumos para la ligadura del cordón umbilical (cinta umbilical, ligadura o equivalente)	( )	( )		
4.16	Equipo para aplicación de las vacunas (jeringas, agujas, cartillas, etc.)	( )	( )		
4.17	Catéter umbilical de 3.5 F y 5 F, disponible en el área de atención al recién nacido	( )	( )		
4.18	¿Cuenta con prueba de tamiz neonatal?	( )	( )	**Número:**	
4.19	Surfactante para los recién nacidos prematuros en la unidad hospitalaria	( )	( )	**Número:**	

**Observaciones de la sección 4 (Especifique el número de pregunta y el comentario):**

**5. Utilización de Servicios**		
**Información estadística y epidemiológica**		**Número:**
5.1	**Total, de mujeres registradas en el CENSO de embarazadas en los <u>últimos 6 meses</u>. Especifique por meses vencidos.** (Se puede encontrar la información en las libretas)	
5.2	**Total, de mujeres primera consulta prenatal en los últimos 6 meses. Especifique por meses vencidos.** (Se puede encontrar la información en las libretas)	
5.3	**¿Cuántos partos se han atendido en los últimos seis meses?**	
5.3.1	Consultar al personal de ginecología: ¿Cuánto es el tiempo promedio de estancia post-parto natural?	
5.4	**¿Cuántos cesáreas se han atendido en los últimos seis meses?**	
5.4.1	Consultar al personal de ginecología: ¿Cuánto es el tiempo promedio de estancia post-cesárea?	
5.5	**¿Cuántos abortos se han atendido en los últimos seis meses?**	
5.6	**¿Cuántos legrados se han atendido en los últimos seis meses?**	
5.7	**¿Cuántos AMEUS se han realizado en los últimos seis meses?**	
5.8	**¿Cuántos casos de preeclampsia se diagnosticaron en los últimos seis meses?**	
5.8.1	**¿Cuántos casos se refirieron en los últimos seis meses?**	
*Si no hubo casos en los 6 meses previos **pase a 5.9***<br>*Si se registraron máximo 10 casos por favor revise todos los expedientes en donde ocurrió el evento y complete la información solicitada en 5.8.2-5.8.4*		
5.8.2	Número de **<u>expedientes</u>** revisados con diagnóstico de **<u>preeclampsia</u>**	
5.8.3	De los expedientes revisados con diagnóstico preeclampsia ¿Cuántos recibieron **<u>Sulfato de Magnesio</u>** (SO_4Mg) durante el tratamiento?	
5.8.3	**<u>Número ámpulas</u>** administradas de (**SO_4Mg**) durante el tratamiento (Total de ámpulas)	
5.9	¿Cuántos casos de eclampsia se diagnosticaron y se refirieron en los últimos seis meses?	
5.9.1	¿Cuántos casos de eclampsia se refirieron en los últimos seis meses?	
*(Si no hubo casos en los 6 meses previos **pase a 5.7)***<br>*Si se registraron casos por favor revise todos los expedientes en donde ocurrió el evento y complete la información solicitada en 5.9.2-5.9.4*		
5.9.2	**<u>Número de expedientes</u>** revisados con diagnóstico de **<u>eclampsia</u>**	
5.9.3	De los **<u>expedientes revisados</u>** con diagnóstico eclampsia ¿Cuántos recibieron Sulfato de Magnesio (**SO4Mg**) durante el tratamiento?	
5.9.4	**<u>Número gramos</u>** administrados de (**SO_4Mg**) durante el tratamiento:	

	Expediente	Dosis (gr)
	1	
	2	
	3	
	4	
	5	
	6	
	7	
	8	
	9	
	10	
5.10	¿Cuántas casos de preeclampsia evolucionaron en eclampsia en los últimos seis meses?	
5.11	¿Cuántos casos de hemorragia obstétrica se han atendido en los últimos seis meses?	
5.12	¿Cuántos casos de hemorragias obstétricas se han referido a algún hospital resolutivo en los últimos seis meses?	
5.13	¿Cuántas transfusiones se han realizado por hemorragia obstétrica en los últimos seis meses?	
5.14	¿Cuántas histerectomías obstétricas se han realizado en los últimos seis meses?	
5.15	*** Si existe unidad de Cuidados intensivos, responda, : ¿Cuántos casos obstétricos se han admitido en la unidad de cuidados intensivos en los últimos seis meses? Caso contrario pase a la pregunta 5.16	
5.16	¿Cuántos casos de complicaciones neonatales se han admitido en la unidad de cuidados intensivos en los últimos seis meses?	
5.17	Número de casos en los que se tuvo APGAR menor a 7 a los 5 min. en los últimos seis meses	
5.18	¿Cuántos casos de muertes maternas se han presentado en los últimos seis meses?	

*(Si no hubo casos en los 6 meses previos **pase a 5.19**)*

5.18.1	Especifique *las* ***causas*** de cada una de las ***muertes maternas*** de acuerdo a los códigos de diagnóstico del CIE-10. Usar los certificados de defunción. (Por ejemplo: aborto, hemorragia obstétrica, preeclampsia y eclampsia)	
	No.	**Diagnóstico**
	1	
	2	
	3	
	4	
	5	
	6	
	7	
	8	
	9	

	10	
5.19	¿Cuántos casos de muertes perinatales se han presentado en los últimos seis meses? ***(Se definen muertes perinatales las que se presentan desde el nacimiento hasta el día 28)*** *> Si no hubo casos en los 6 meses previos* ***pase a 5.20***	
5.19.1	Especifique las causas de las muertes perinatales (Muertes desde el nacimiento hasta el día 28) que se han presentado durante los últimos seis meses de acuerdo a los códigos de diagnóstico del CIE-10. Usar los certificados de defunción. Revisar mínimo 10 expedientes. (Si hay más de 10, agregar al final del cuestionario)	
	No.	**Diagnóstico**
	1	
	2	
	3	
	4	
	5	
	6	
	7	
	8	
	9	
	10	
5.20	¿Cuántas referencias de mujeres con complicaciones obstétricas se han realizado en los últimos seis meses?	
5.21	¿Cuántas referencias por complicaciones neonatales se han realizado en los últimos seis meses?	
5.22	¿Cuántos casos de óbitos se reportaron en los últimos seis meses?	

5.23 Planeación estratégica

Instrucciones: Esta información puede ser proporcionada por el personal directivo o por los encargados de gestoría y administración

La unidad de salud realiza una estimación de:		**No**	**Si**	**Especificar el dato**
5.23.1	*Tasa de fecundidad*	( )	( )	
5.23.2	*Población beneficiaria en edad fértil*	( )	( )	
5.23.3	*Número esperado de embarazos de bajo riesgo*	( )	( )	
5.23.4	*Número esperado de embarazos complicados*	( )	( )	
5.23.5	*Número esperado de partos de bajo riesgo*	( )	( )	

5.23.6	*Número esperado de partos complicados*			
5.23.7	*Número esperado de recién nacidos*	( )	( )	
5.23.8	*Mujeres hospitalizadas por atención perinatal*	( )	( )	
5.23.9	*Frecuencia de partos pretérmino*	( )	( )	
5.23.10	*Frecuencia de partos postérmino*	( )	( )	
5.23.11	*Promedio de días de estancia en la UCIN*	( )	( )	
5.23.12	*Tasa de infecciones puerperales*	( )	( )	
5.23.13	¿La unidad de salud realiza planeación estratégica de insumos según población en edad fértil?			*Si……………………… ( )*<br>*No…………………… ( )*
5.23.14	¿Hay evaluación de los sistemas de distribución de insumos dentro de la unidad hospitalaria?			*Si……………………… ( )*<br>*No…………………… ( )*
5.23.15	¿Hay evaluación de los sistemas de distribución de medicamentos dentro de la unidad hospitalaria?			*Si……………………… ( )*<br>*No…………………… ( )*

**Observaciones de la sección 5 (Especifique el número de pregunta y el comentario):**

Referencias:

1. NORMA Oficial Mexicana NOM-007-SSA2-2016, Para la atención de la mujer durante el embarazo, parto y puerperio, y de la persona recién nacida.

2. Guía de Práctica clínica. Vigilancia y manejo del trabajo de parto en embarazo de bajo riesgo. México: Secretaría de Salud; 11 de diciembre de 2014.

3. Berdichevsky, K., Diaz-Olavarrieta, C., McCarthy, K., and Blanc, A. 2014. “Validating Indicators of the Quality of Maternal Health Care: Final Report, Mexico.” Mexico City: Population Council.

4. Instituto Nacional de Salud Pública y Comité Promotor por una Maternidad Segura en México. Resultados 1er Taller: Calidad de la Atención en el embarazo, parto y puerperio (CAEPP). 5 noviembre 2014.

5. Instituto Nacional de Salud Pública. 2º Taller: Calidad de la Atención en el Embarazo, Parto, Puerperio, y del Recién Nacido (CAEPPyRN). 28 enero 2016.

6. Modelo de Recursos para la Planeación de Unidades Médicas de la Secretaría de Salud (Unidad de Parto Humanizado). Dirección General de Planeación y Desarrollo en Salud (DGPLADES), México 2016.

Instituciones colaboradoras y participantes de los Talleres CAEPPyRN:

Centro para los adolescentes de San Miguel de Allende, A.C. (CASA)
Centro de Colaboración Cívica (CCC)
Dirección General de Planeación y Desarrollo en Salud (DGPLADES)
Instituto de Seguridad y Servicios Sociales de los Trabajadores del Estado (ISSSTE)
Instituto Nacional de Salud Pública (INSP)
Secretaria de Salud de Morelos (SSM)
IPAS, México
Comité Promotor por una Maternidad Segura en México (CPMS)
Instituto Mexicano del Seguro Social (IMSS)
Observatorio de Mortalidad Materna (OMM)
K'inal Antzetik, A.C.
Centro Nacional de Equidad de Género y Salud Reproductiva (CNEGySR)
Dirección de Calidad, Servicios de Saud, Veracruz
Centro de Investigaciones y Estudios Superiores en Antropología Social (CIESAS)
Instituto Nacional de Perinatología (INPer)
Hospital General de Tula, Servicios de Salud de Hidalgo (SSH)
Colectivo Maternidad Empoderada (CME)
Consultora Independiente, Grupo de Información en Reproducción Elegida, A.C. (GIRE)
Centro de Investigación Materno Infantil del Grupo de Estudios al Nacimiento (CIMIGEN)
Colectivo de Investigación, Desarrollo y Educación entre Mujeres, A.C. (CIDEM)
FUNDAR, Centro de Análisis e Investigación
World Vision
Save the children
Asociación Mexicana de Partería (AMP)
Luna Maya, Casa de Partos
Balance A.C.
Secretaria de Salud de Durango (SSD)
Hospital General de León, Guanajuato
Parteras Tradicionales Unidas Tumben Cuxtal
Fondo de Población de las Naciones Unidas, México (UNFPA)
United Nations Children's Fund (UNICEF)
Universidad de California, San Francisco (UCSF)
Partners in Health
MacArthur Foundation, México
